# Supplementary material for: Design, Synthesis and Biological Evaluation of Lophanic Acid Derivatives as Antifungal and Antibacterial Agents
Source: Molecules. 2022 Oct 12;27(20):6836. doi: 10.3390/molecules27206836 (PMC9611534; doi:10.3390/molecules27206836)
Supplement: Supplementary file 1 [file molecules-27-06836-s001.zip › molecules-1953236-Supplementary.pdf]

Supplementary material

Design, Synthesis and Biological Evaluation of Lophanic acid derivatives as Antifungal Agents and Antibacterial Agents

Xiang Yu [1,†] , Xun Song [2,3,†], Yi Zhang [1,†], Yemeng Yang [3,†,] Jianghai Ye [1], Yahua Liu [1], Lutai Pan [1,*] and Hongjie Zhang [3,*]

[1] The Key Laboratory of Miao Medicine of Guizhou Province, Guizhou University of Traditional Chinese Medicine, Guiyang 550025, China;

[2] College of Pharmacy, Shenzhen Technology University, Shenzhen 518118, People's Republic of China

[3] School of Chinese Medicine, Hong Kong Baptist University, Hong Kong, People's Republic of China.

[†]These authors contributed equally to this work.

[*]Corresponding Author: Lutai Pan, Email: ltpan@sina.cn;
Hongjie Zhang, Email: zhanghj@hkbu.edu.h

Contents

Figure S1. ^{1}H NMR spectrum of the compound **3a**.

Figure S2. ^{13}C NMR spectrum of the compound **3a**.

Figure S3. HRMS spectrum of the compound **3a**.

Figure S4. ^{1}H NMR spectrum of the compound **3b**.

Figure S5. ^{13}C NMR spectrum of the compound **3b**.

Figure S6. HRMS spectrum of the compound **3b**.

Figure S7. ^{1}H NMR spectrum of the compound **3c**.

Figure S8. ^{13}C NMR spectrum of the compound **3c**.

Figure S9. HRMS spectrum of the compound **3c**.

Figure S10. ^{1}H NMR spectrum of the compound **3d**.

Figure S11. ^{13}C NMR spectrum of the compound **3d**.

Figure S12. HRMS spectrum of the compound **3d**.

Figure S13. ^{1}H NMR spectrum of the compound **3e**.

Figure S14. ^{13}C NMR spectrum of the compound **3e**.

Figure S15. HRMS spectrum of the compound **3e**.

Figure S16. ^{1}H NMR spectrum of the compound **3f**.

Figure S17. ^{13}C NMR spectrum of the compound **3f**.

Figure S18. HRMS spectrum of the compound **3f**.

Figure S19. ^{1}H NMR spectrum of the compound **3g**.

Figure S20. ^{13}C NMR spectrum of the compound **3g**.

Figure S21. HRMS spectrum of the compound **3g**.

Figure S22. ^{1}H NMR spectrum of the compound **3h**.

Figure S23. ^{13}C NMR spectrum of the compound **3h**.

Figure S24. HRMS spectrum of the compound **3h**.

Figure S25. ^{1}H NMR spectrum of the compound **3i**.

Figure S26. ^{13}C NMR spectrum of the compound **3i**.

Figure S27. HRMS spectrum of the compound **3i**.

Figure S28. ^{1}H NMR spectrum of the compound **3j**.

Figure S29. ^{13}C NMR spectrum of the compound **3j**.

Figure S30. HRMS spectrum of the compound **3j**.

Figure S31. ^{1}H NMR spectrum of the compound **3k**.

Figure S32. ^{13}C NMR spectrum of the compound **3k**.

Figure S33. HRMS spectrum of the compound **3k**.

Figure S34. ^{1}H NMR spectrum of the compound **3l**.

Figure S35. ^{13}C NMR spectrum of the compound **3l**.

Figure S36. HRMS spectrum of the compound **3l**.

Figure S37. ^{1}H NMR spectrum of the compound **3m**.

Figure S38. ^{13}C NMR spectrum of the compound **3m**.

Figure S39. HRMS spectrum of the compound **3m**.

Figure S40. ^{1}H NMR spectrum of the compound **3n**.

Figure S41. ^{13}C NMR spectrum of the compound **3n**.

Figure S42. HRMS spectrum of the compound **3n**.

Figure S43. ^{1}H NMR spectrum of the compound **4**.

Figure S44. ^{13}C NMR spectrum of the compound **4**.

Figure S45. HRMS spectrum of the compound **4**.

Figure S46. ^{1}H NMR spectrum of the compound **5a**.

Figure S47. ^{13}C NMR spectrum of the compound **5a**.

Figure S48. HRMS spectrum of the compound **5a**.

Figure S49. ^{1}H NMR spectrum of the compound **5b**.

Figure S50. ^{13}C NMR spectrum of the compound **5b**.

Figure S51. HRMS spectrum of the compound **5b**.

Figure S52. ^{1}H NMR spectrum of the compound **5c**.

Figure S53. ^{13}C NMR spectrum of the compound **5c**.

Figure S54. HRMS spectrum of the compound **5c**.

Figure S55. ^{1}H NMR spectrum of the compound **5d**.

Figure S56. ^{13}C NMR spectrum of the compound **5d**.

Figure S57. HRMS spectrum of the compound **5d**.

Figure S58. ^{1}H NMR spectrum of the compound **5e**.

Figure S59. ^{13}C NMR spectrum of the compound **5e**.

Figure S60. HRMS spectrum of the compound **5e**.

Figure S61. ^{1}H NMR spectrum of the compound **5f**.

Figure S62. ^{13}C NMR spectrum of the compound **5f**.

Figure S63. HRMS spectrum of the compound **5f**.

Figure S64. ^{1}H NMR spectrum of the compound **5g**.

Figure S65. ^{13}C NMR spectrum of the compound **5g**.

Figure S66. HRMS spectrum of the compound **5g**.

Figure S67. ^{1}H NMR spectrum of the compound **5h**.

Figure S68. ^{13}C NMR spectrum of the compound **5h**.

Figure S69. HRMS spectrum of the compound **5h**.

Figure S70. ^{1}H NMR spectrum of the compound **6a**.

Figure S71. ^{13}C NMR spectrum of the compound **6a**.

Figure S72. HRMS spectrum of the compound **6a**.

Figure S73. ^{1}H NMR spectrum of the compound **6b**.

Figure S74. ^{13}C NMR spectrum of the compound **6b**.

Figure S75. HRMS spectrum of the compound **6b**.

Figure S76. ^{1}H NMR spectrum of the compound **6c**.

Figure S77. ^{13}C NMR spectrum of the compound **6c**.

Figure S78. HRMS spectrum of the compound **6c**.

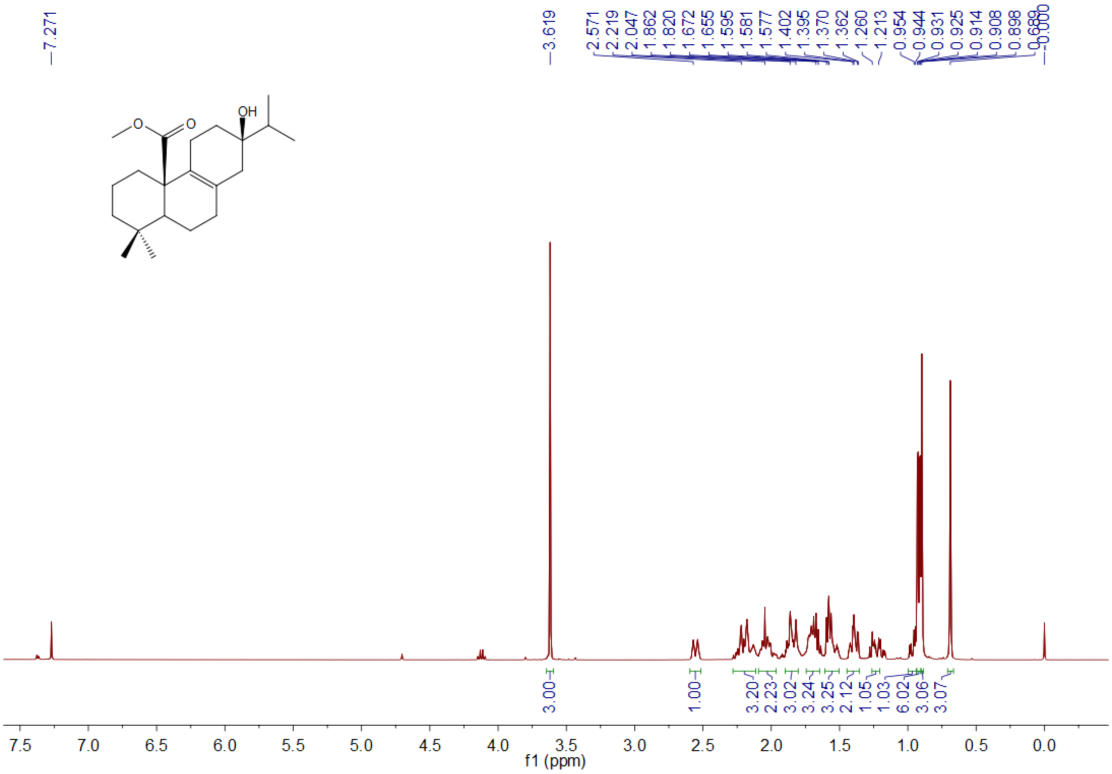

Figure S1. ^{1}H NMR spectrum of the compound **3a**.

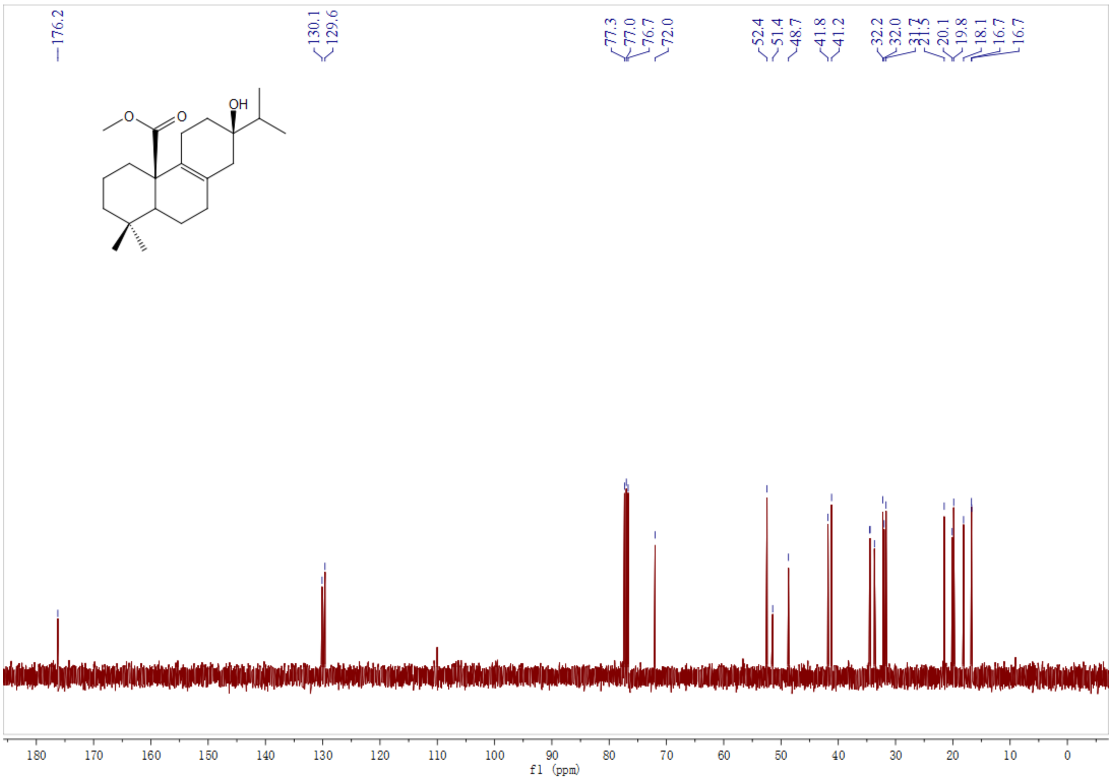

Figure S2. ^{13}C NMR spectrum of the compound **3a**.

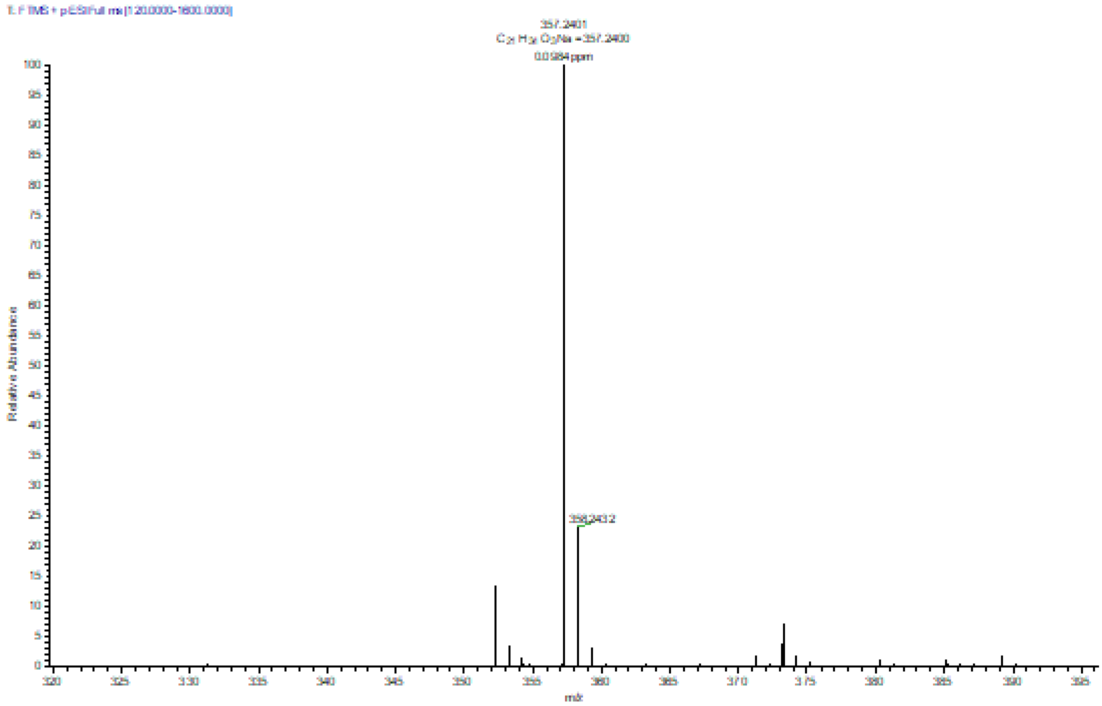

Figure S3. HRMS spectrum of the compound **3a**.

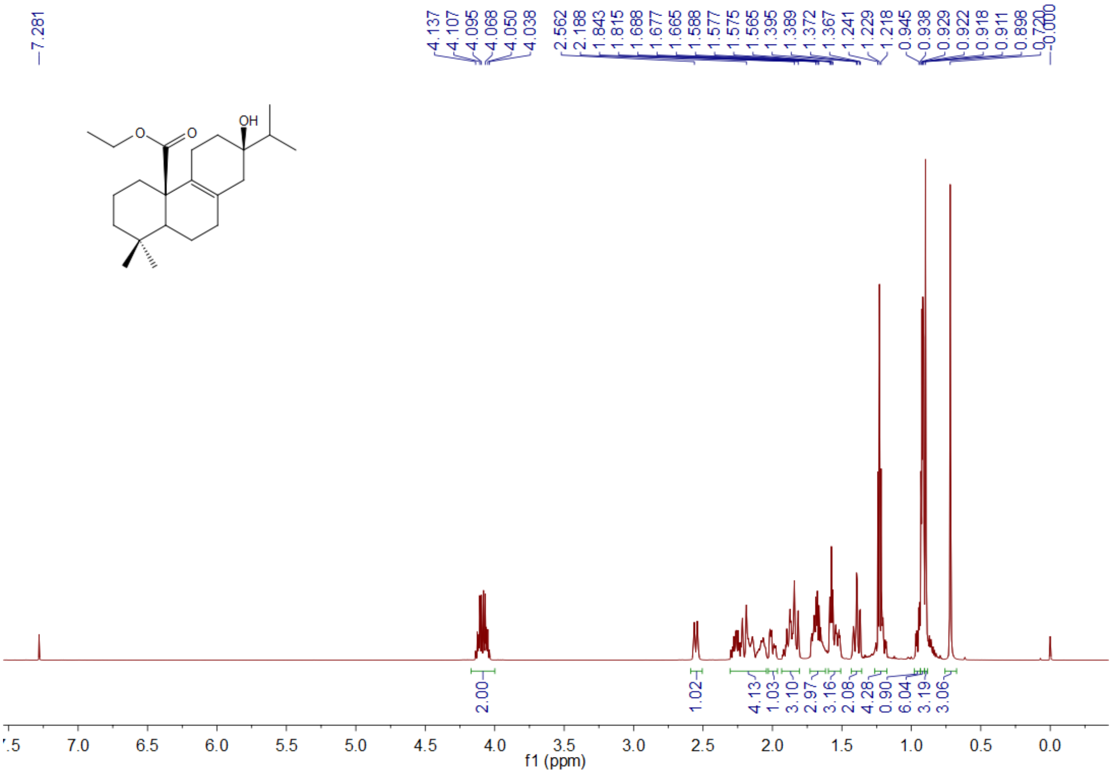

Figure S4. ^{1}H NMR spectrum of the compound **3b**.

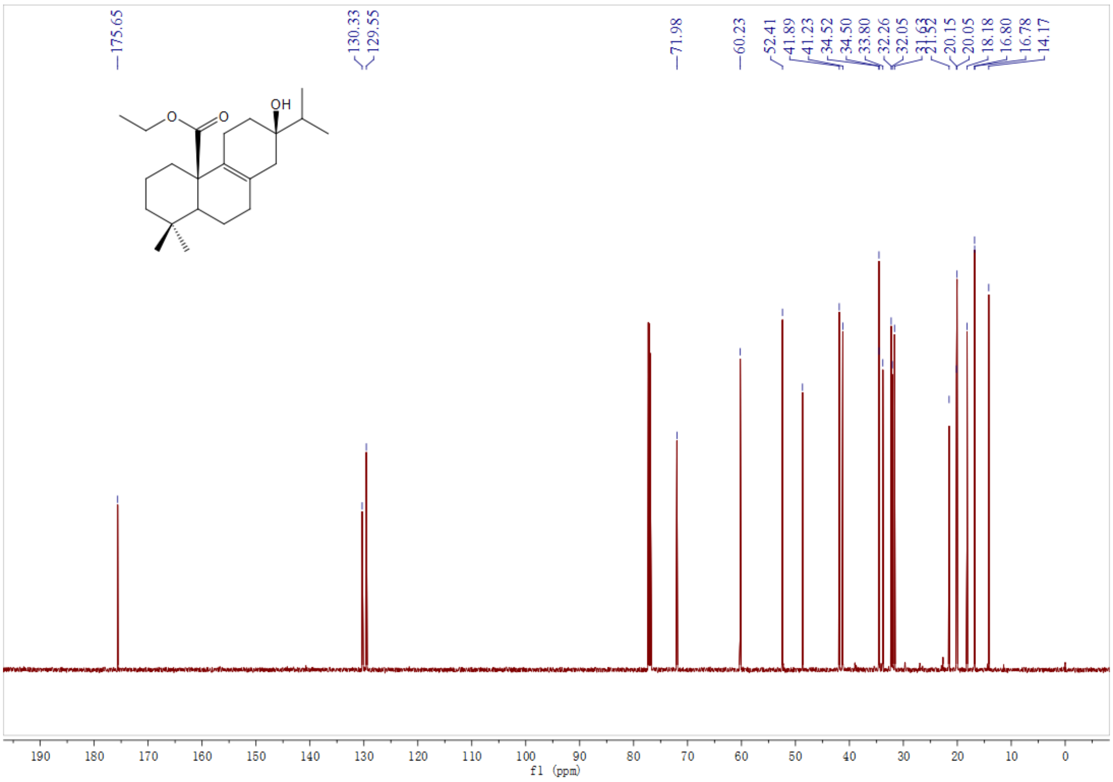

Figure S5. ^{13}C NMR spectrum of the compound **3b**.

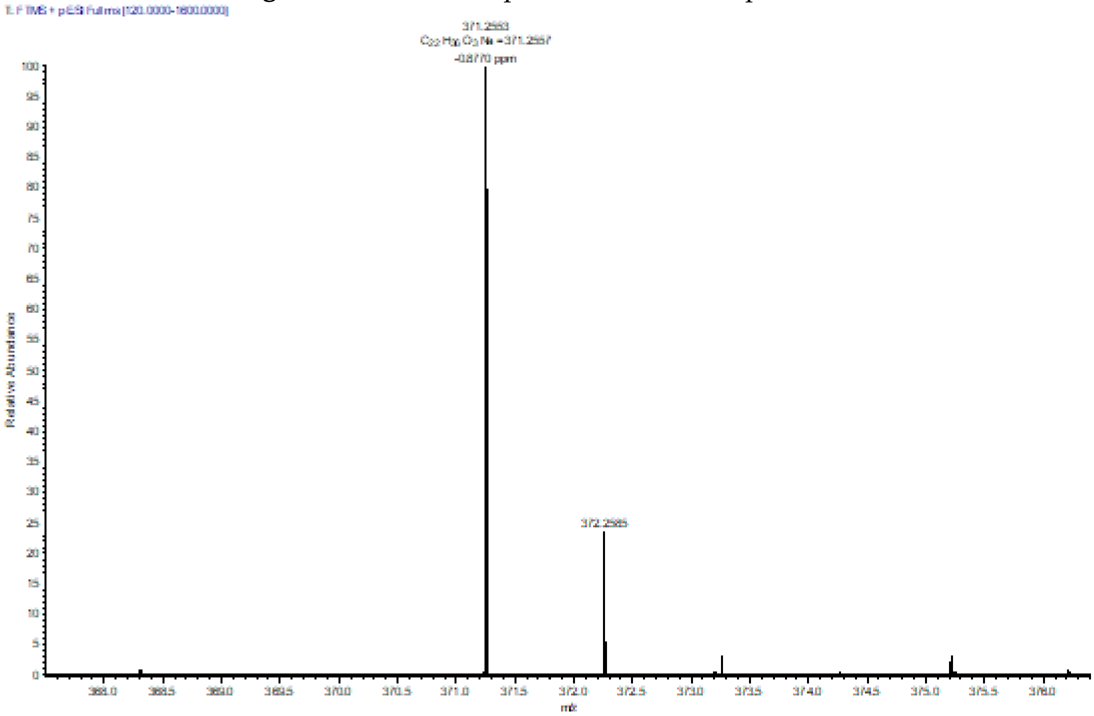

Figure S6. HRMS spectrum of the compound **3b**.

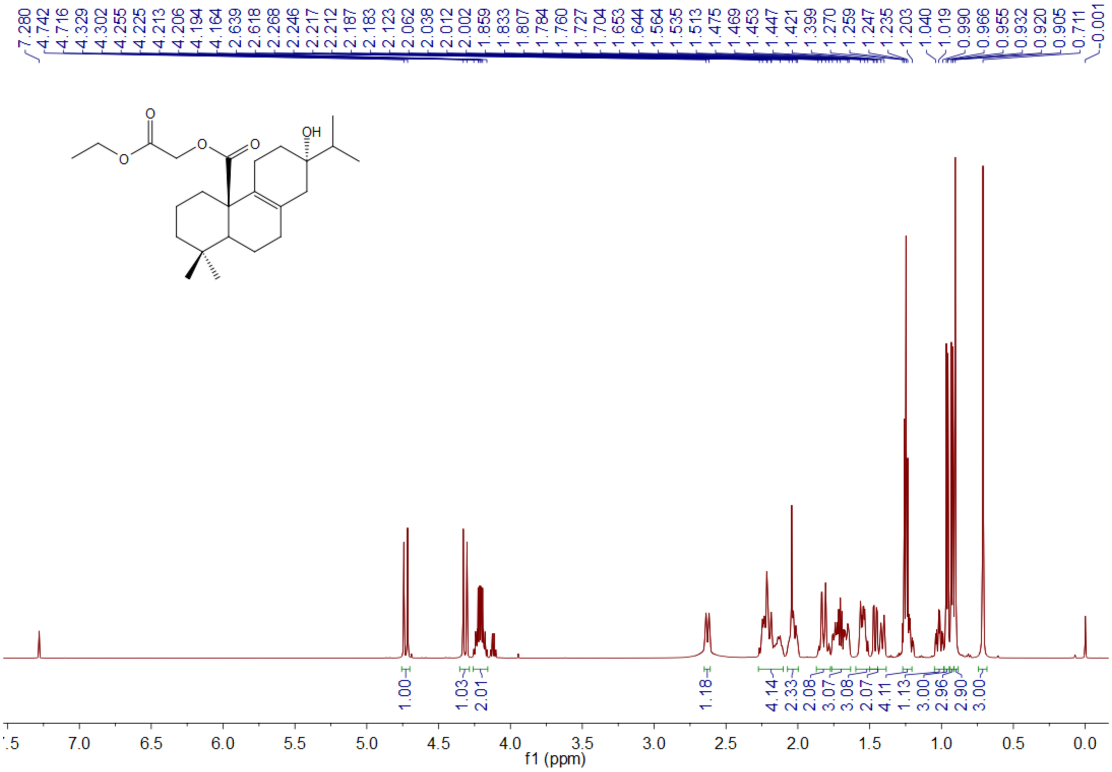

Figure S7. ^{1}H NMR spectrum of the compound **3c**.

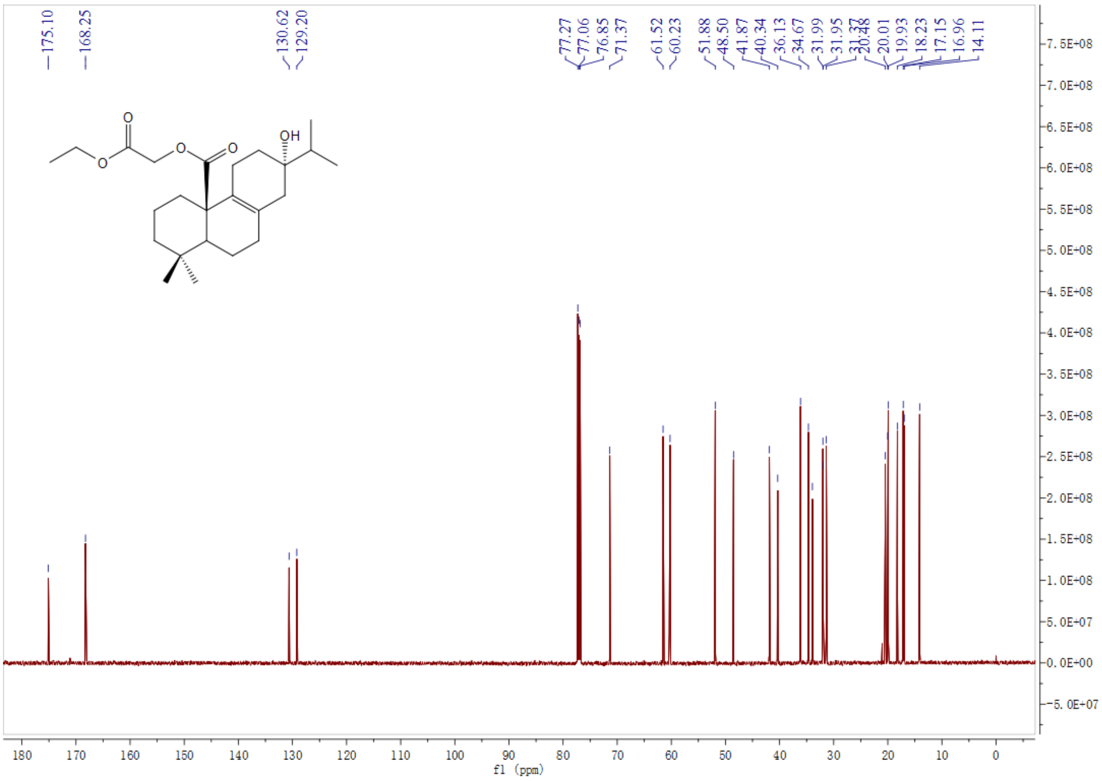

Figure S8. ^{13}C NMR spectrum of the compound **3c**.

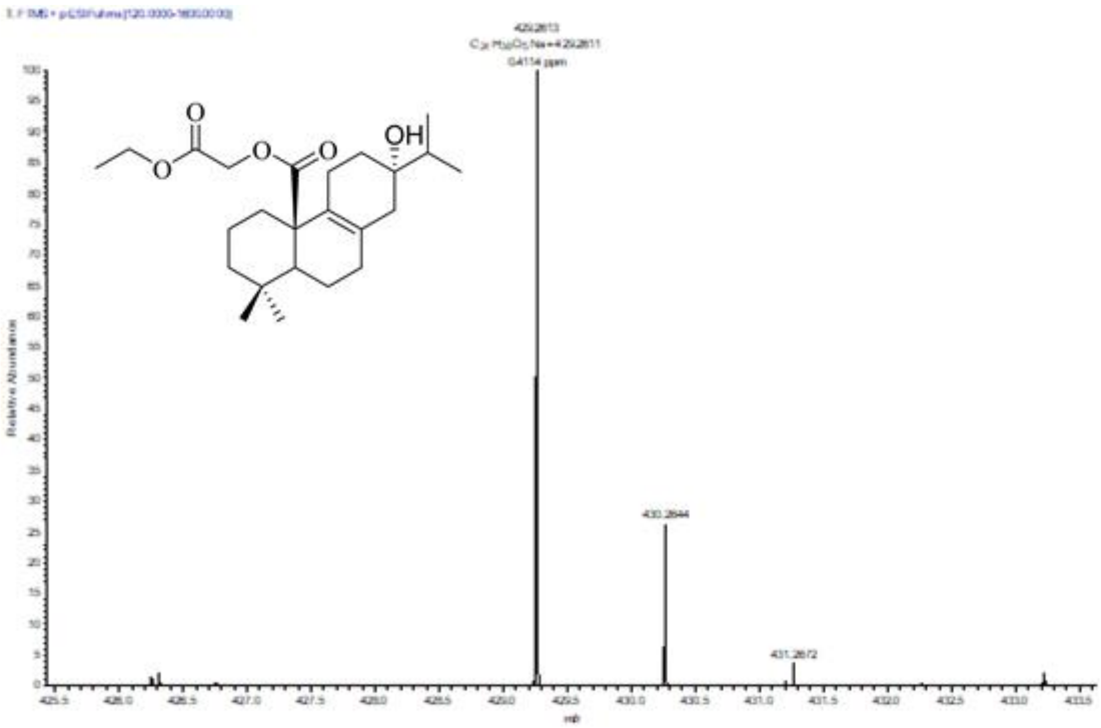

Figure S9. HRMS spectrum of the compound **3c**.

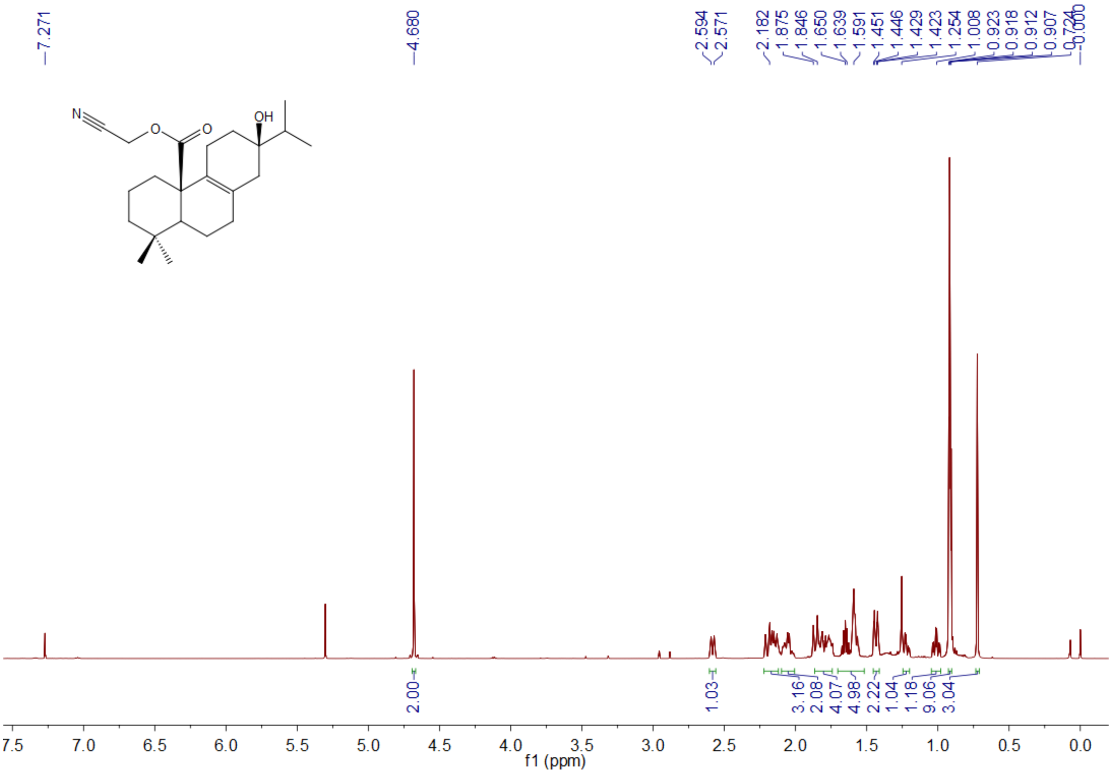

Figure S10. ^{1}H NMR spectrum of the compound **3d**.

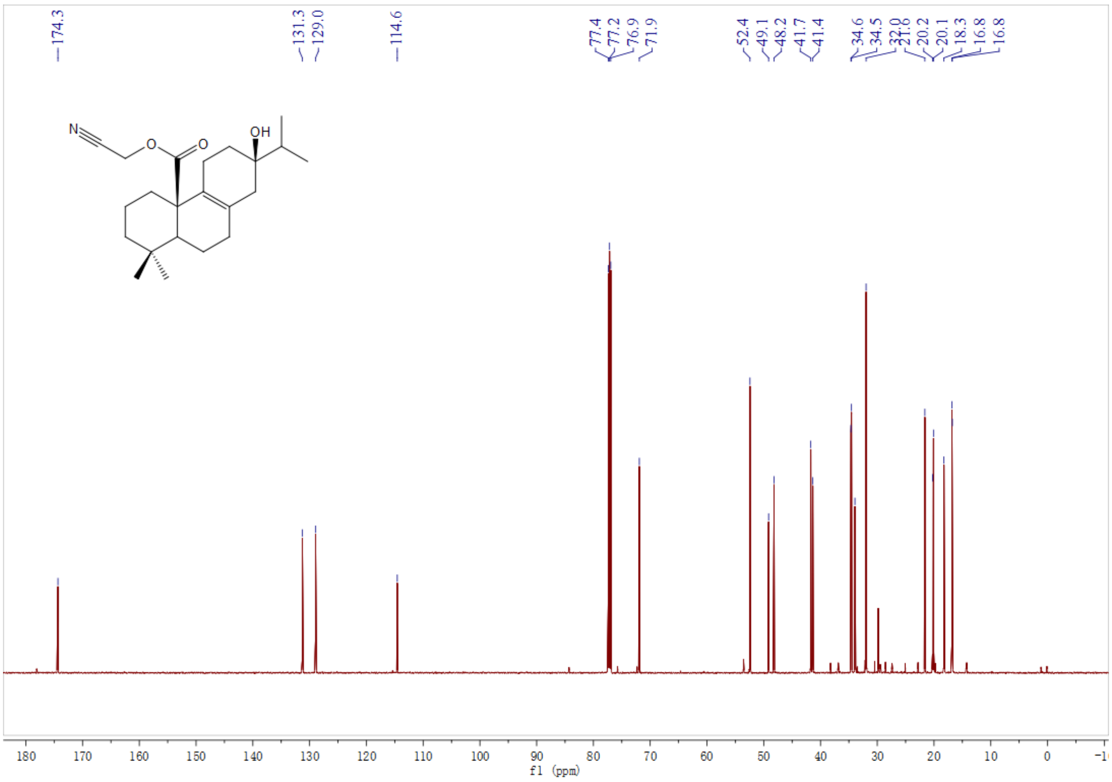

Figure S11. ^{13}C NMR spectrum of the compound **3d**.

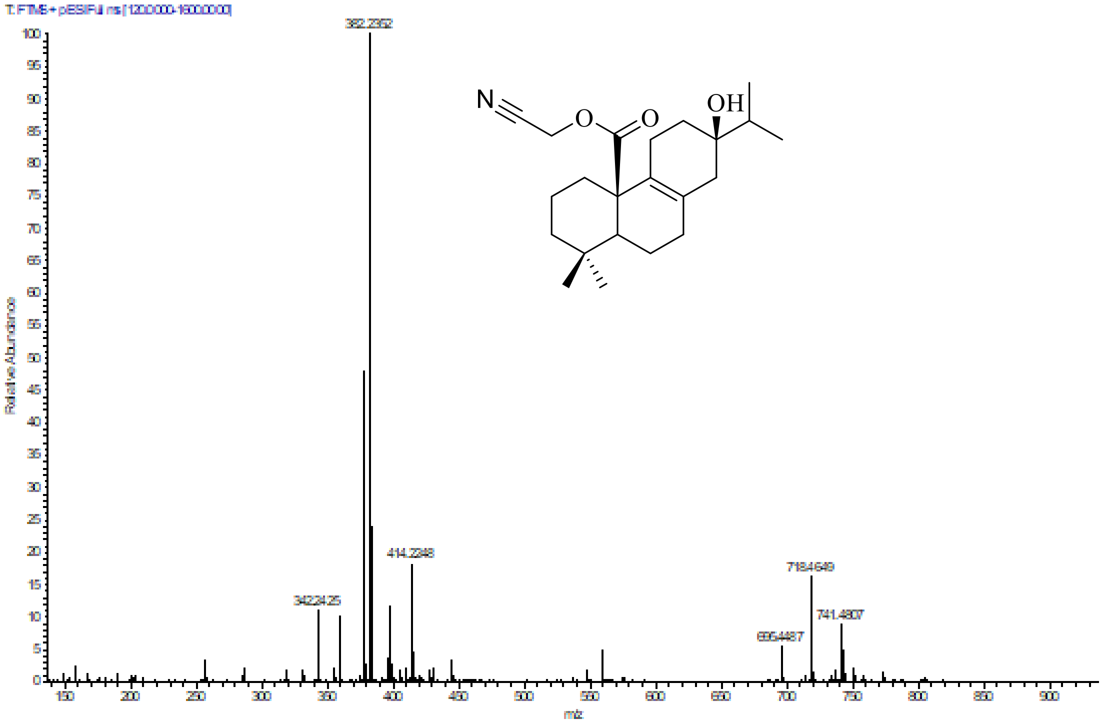

Figure S12. HRMS spectrum of the compound **3d**.

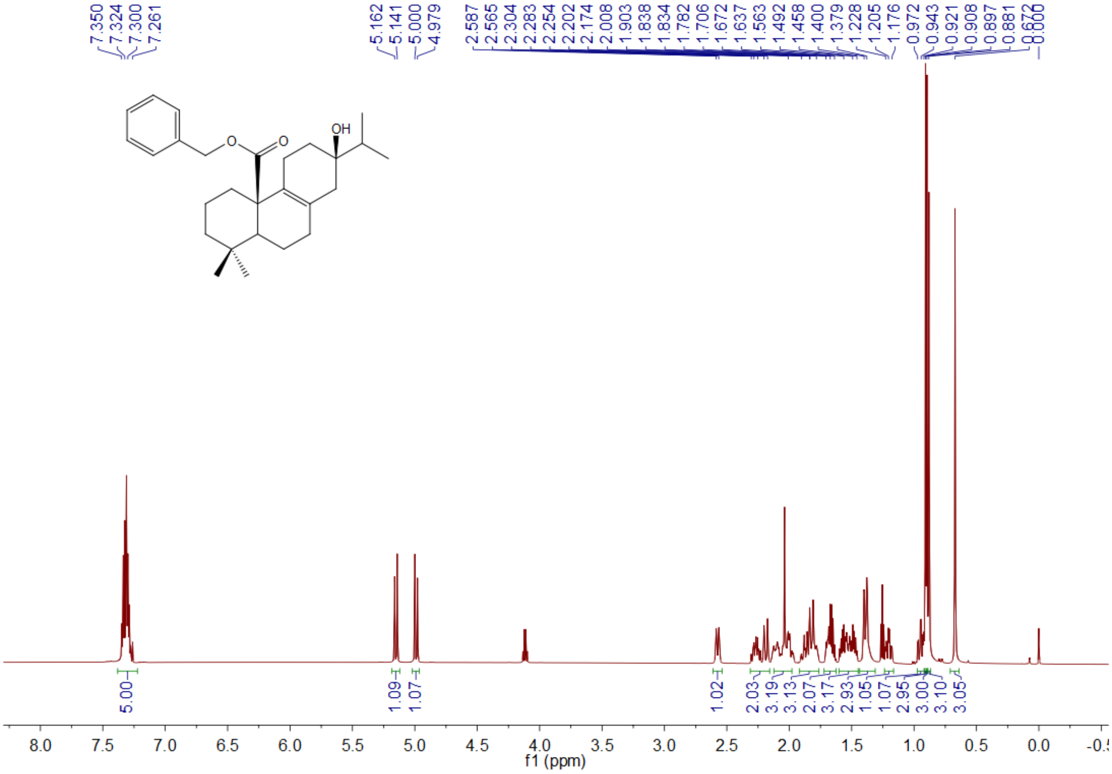

Figure S13. ^{1}H NMR spectrum of the compound **3e**.

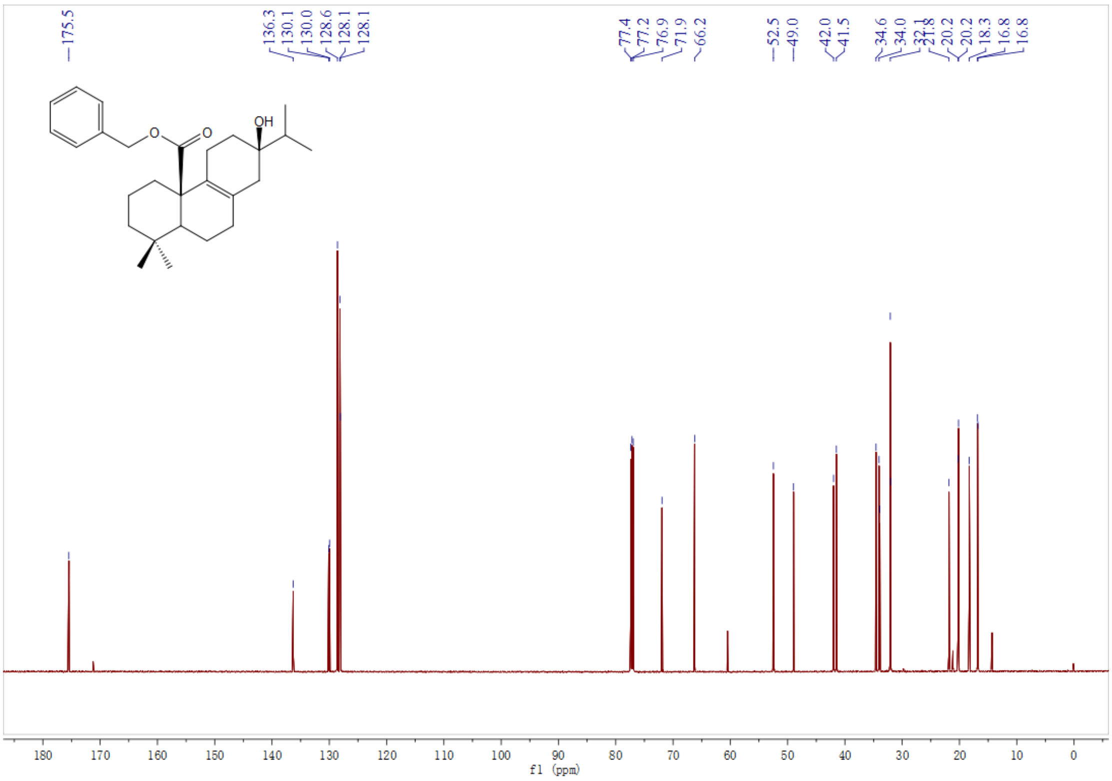

Figure S14. ^{13}C NMR spectrum of the compound **3e**.

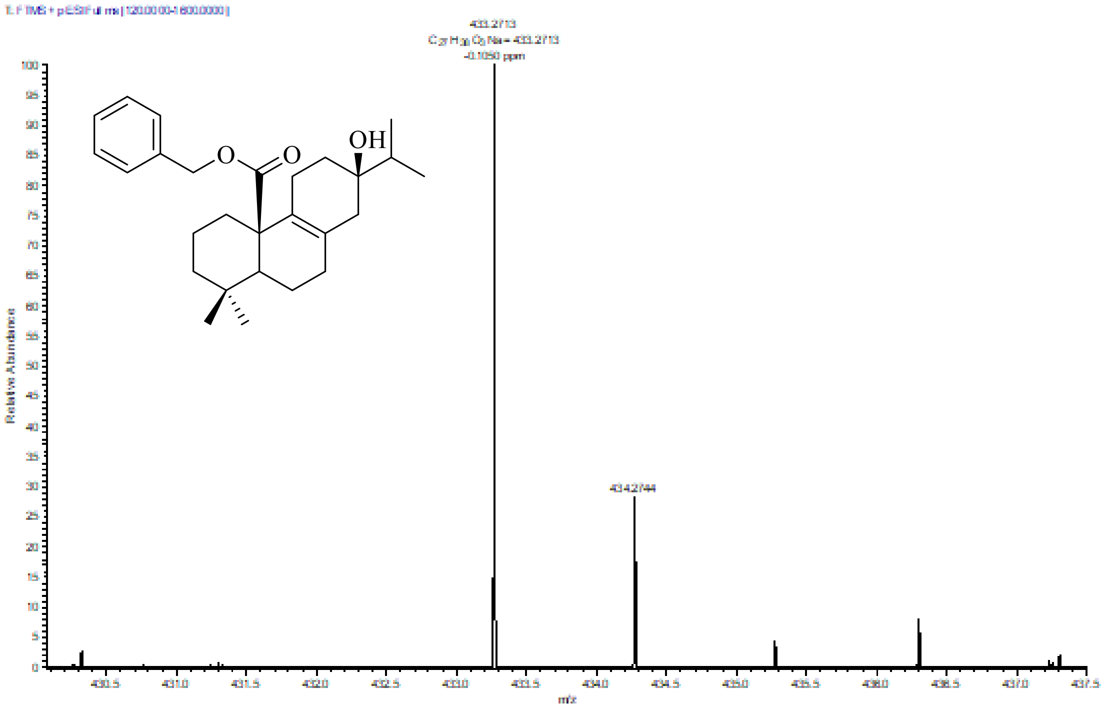

Figure S15. HRMS spectrum of the compound **3e**.

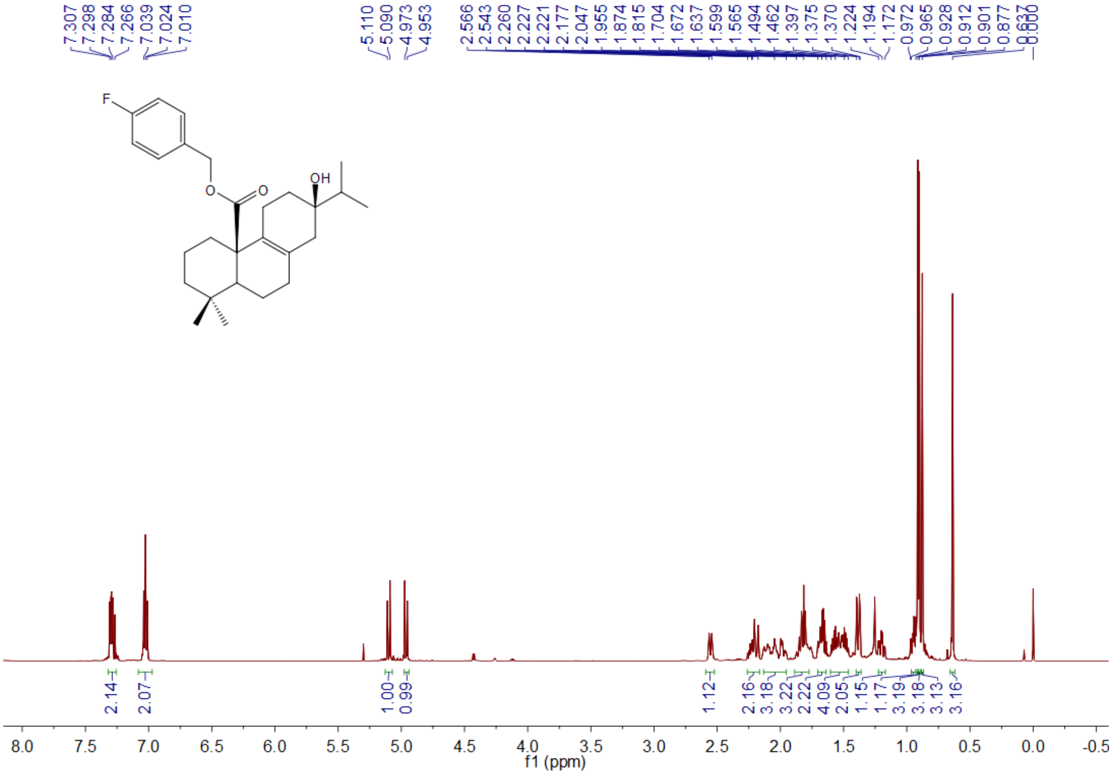

Figure S16. ^{1}H NMR spectrum of the compound **3f**.

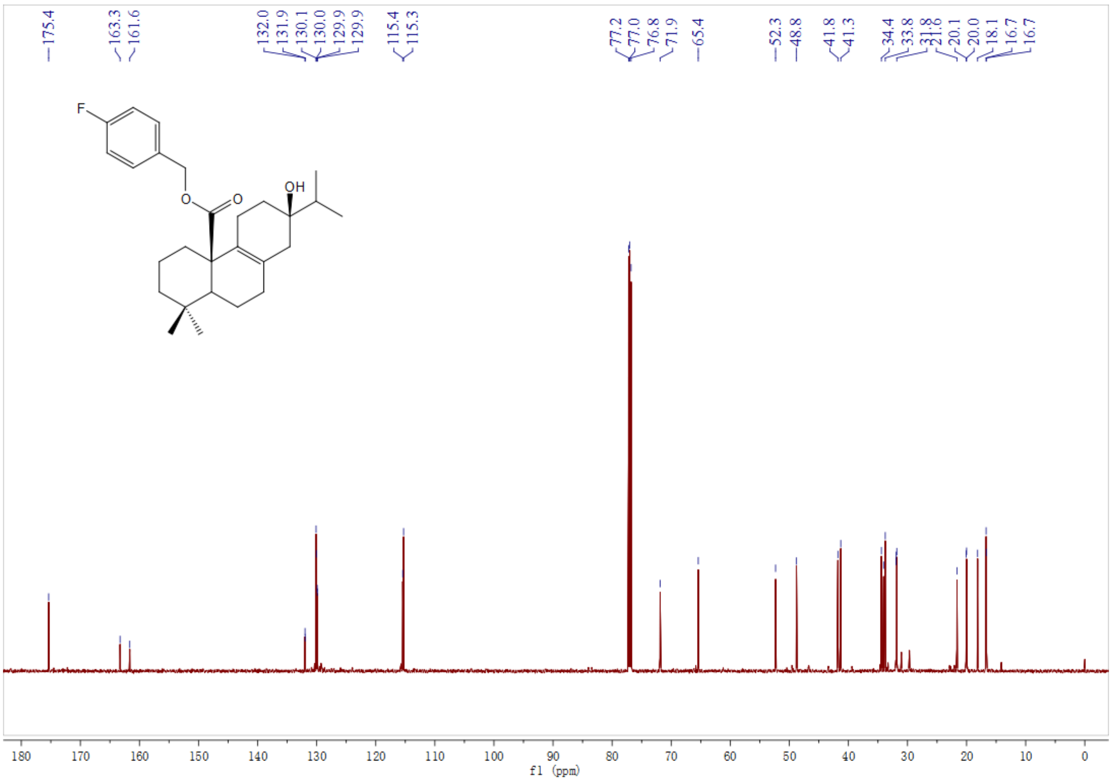

Figure S17. ^{13}C NMR spectrum of the compound **3f**.

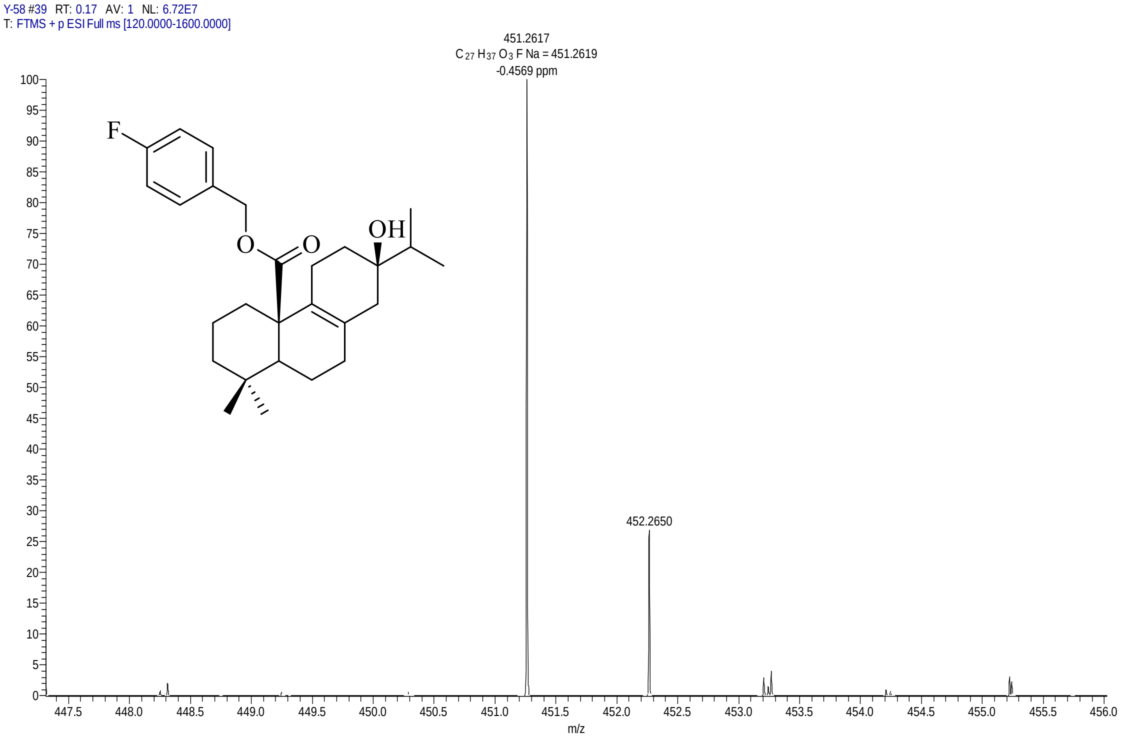

Figure S18. HRMS spectrum of the compound **3f**.

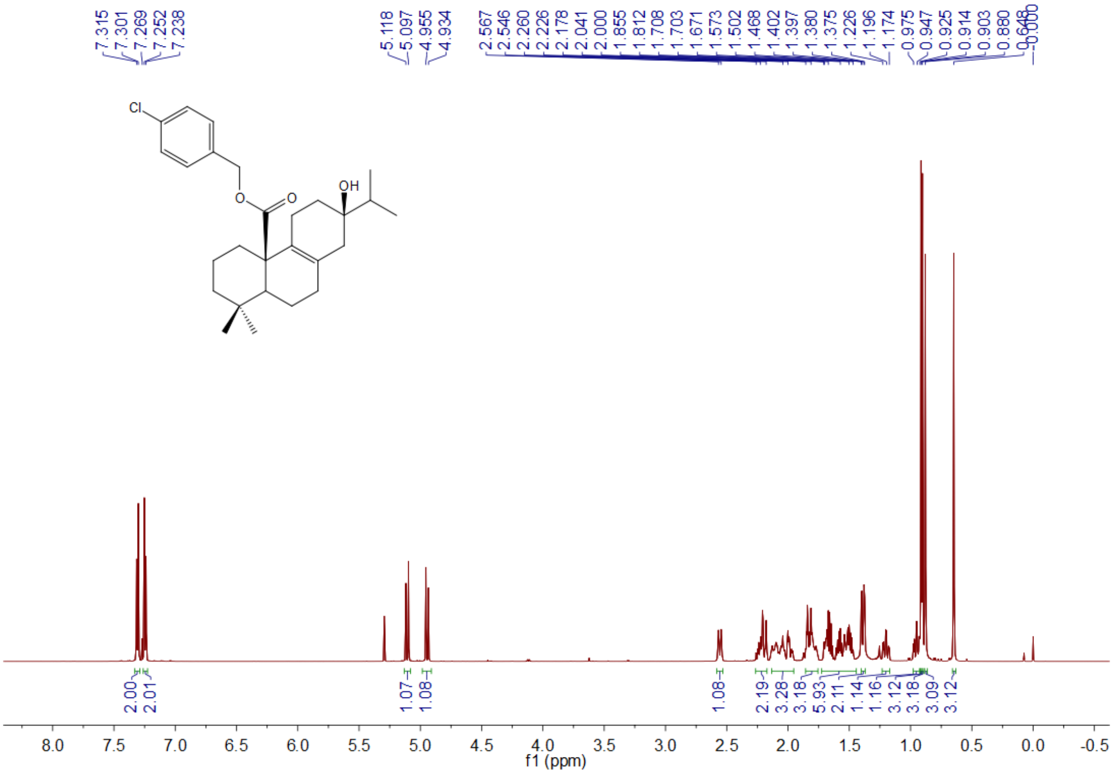

Figure S19. ^{1}H NMR spectrum of the compound **3g**.

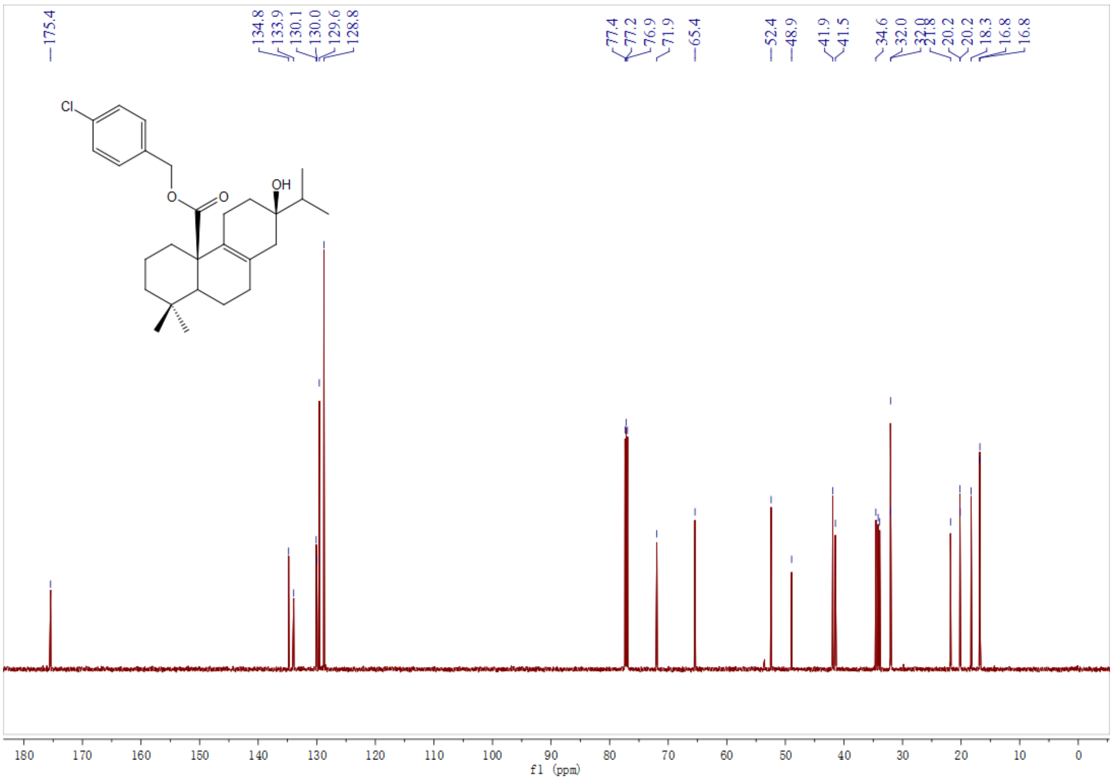

Figure S20. ^{13}C NMR spectrum of the compound **3g**.

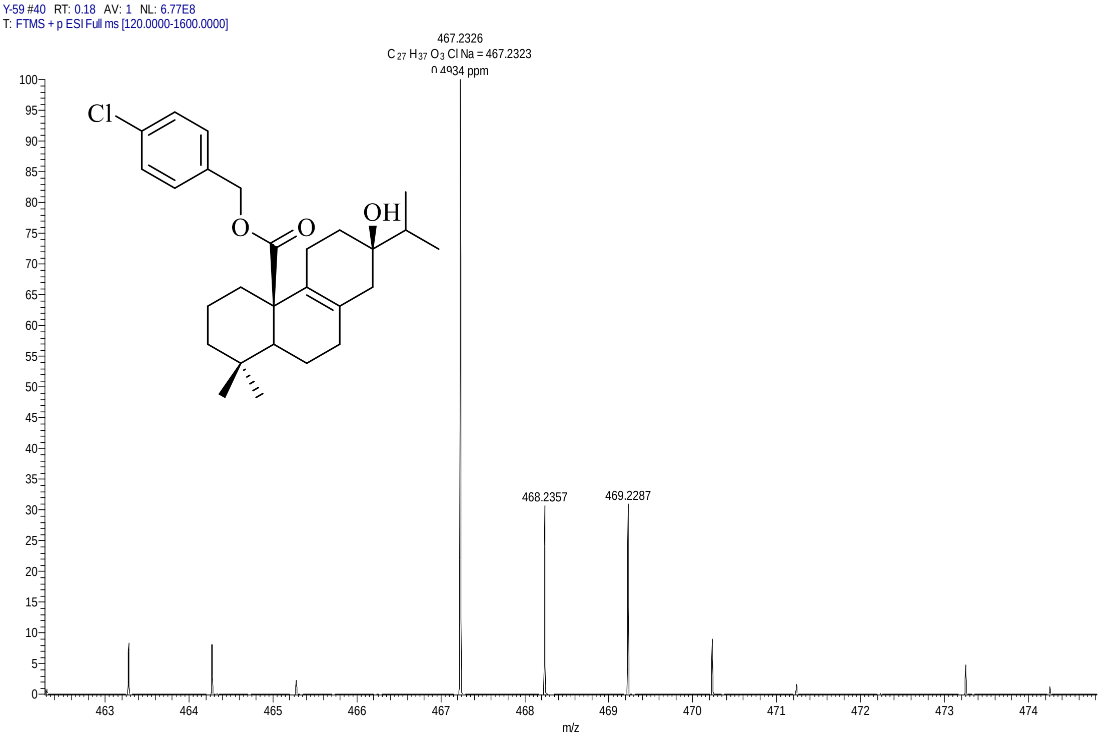

Figure S21. HRMS spectrum of the compound **3g**.

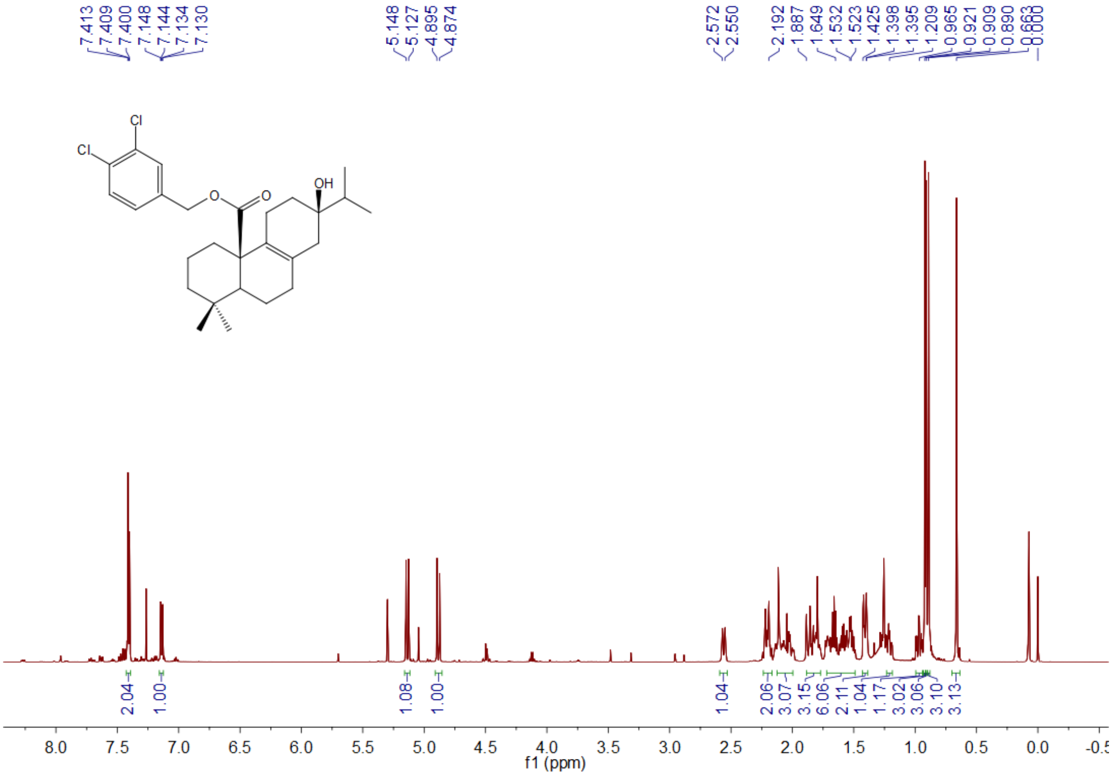

Figure S22. ^{1}H NMR spectrum of the compound **3h**.

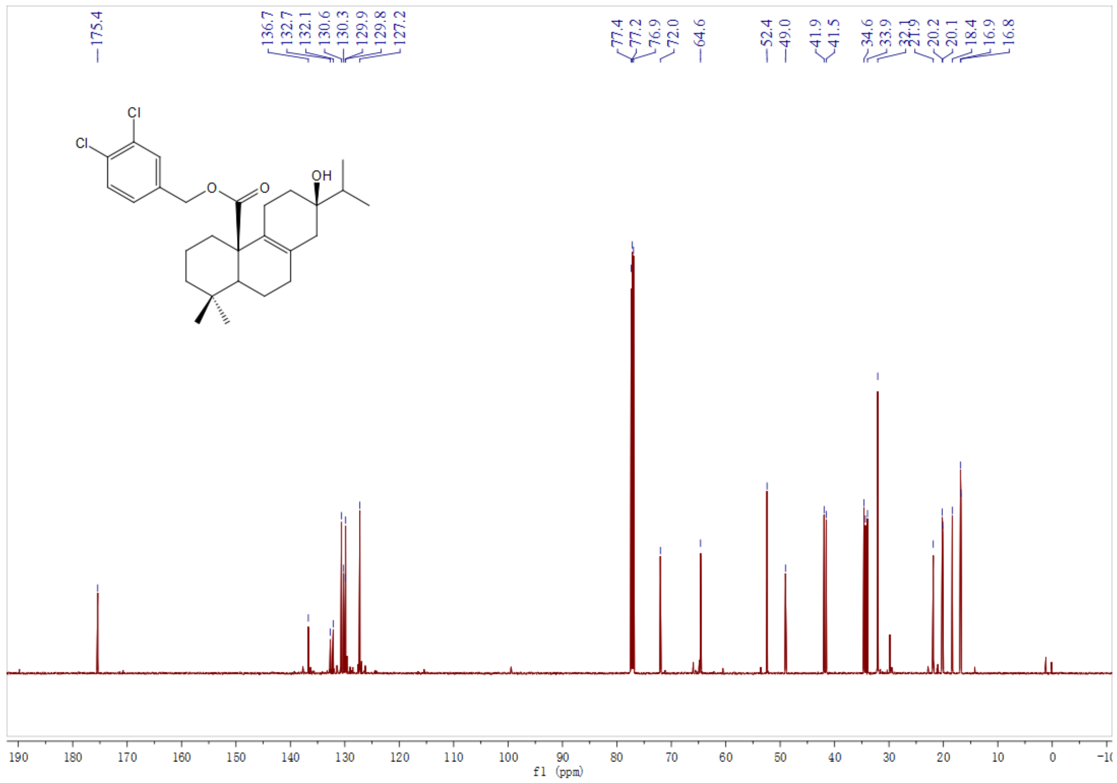

Figure S23. ^{13}C NMR spectrum of the compound **3h**.

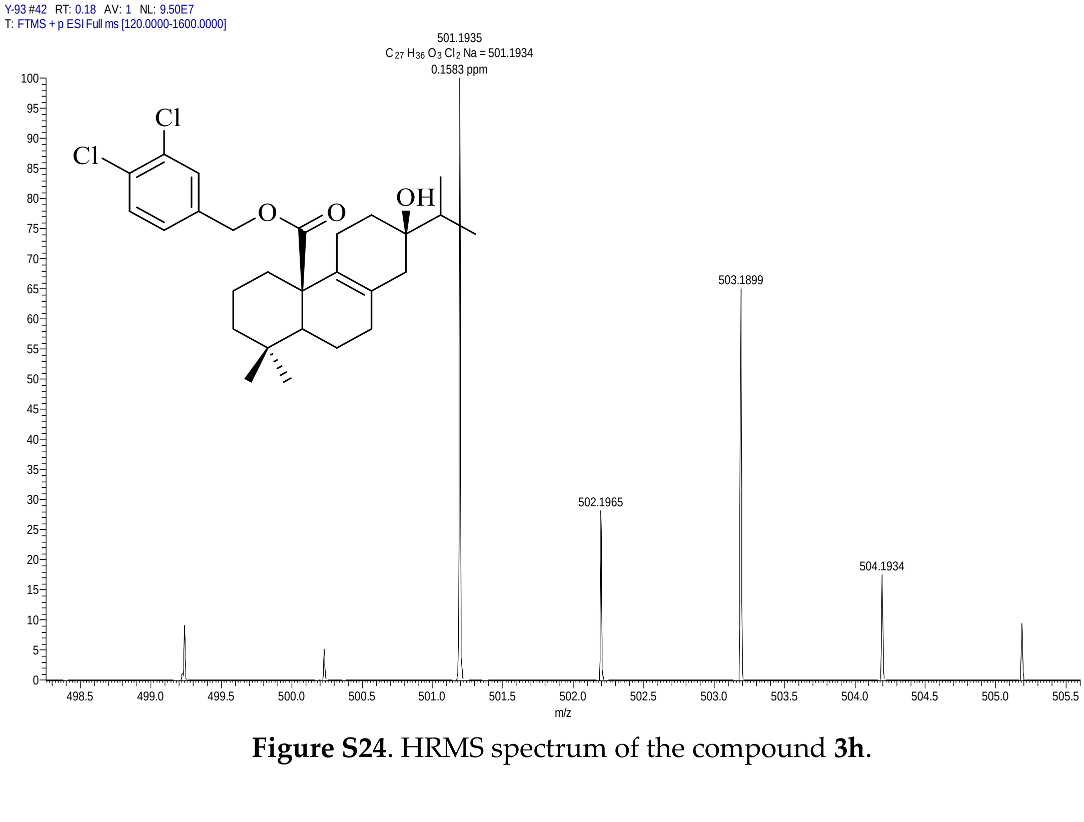

Figure S24. HRMS spectrum of the compound **3h**.

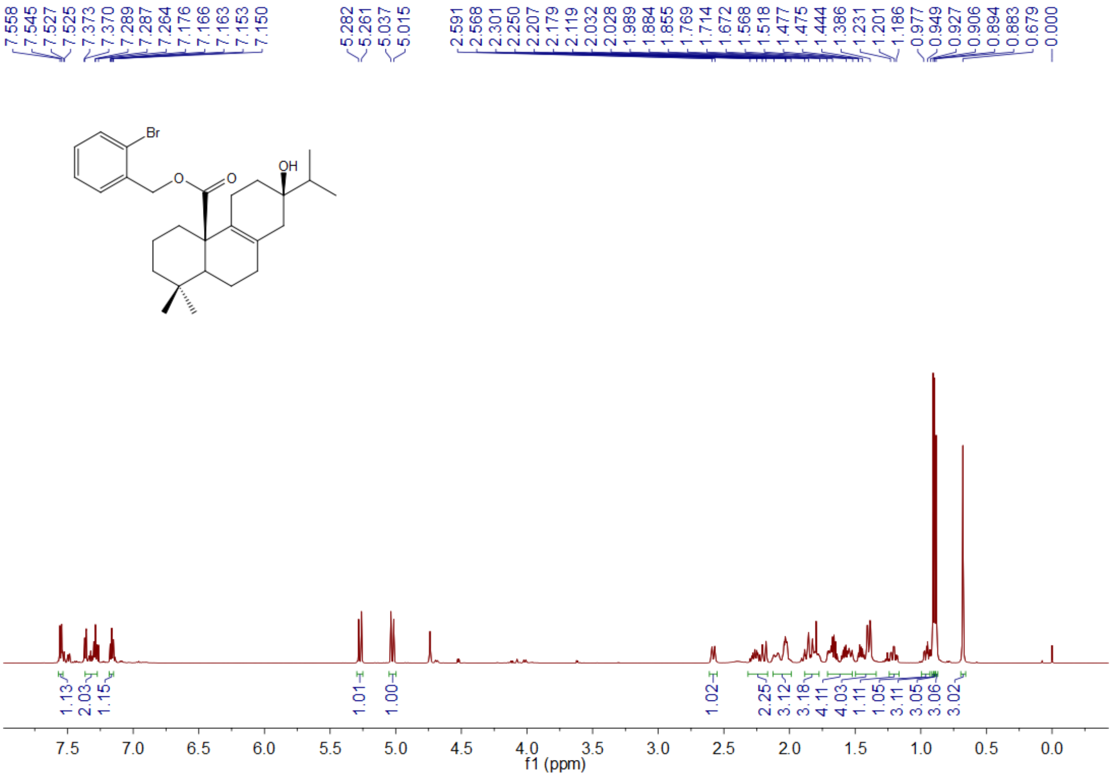

Figure S25. ^{1}H NMR spectrum of the compound **3i**.

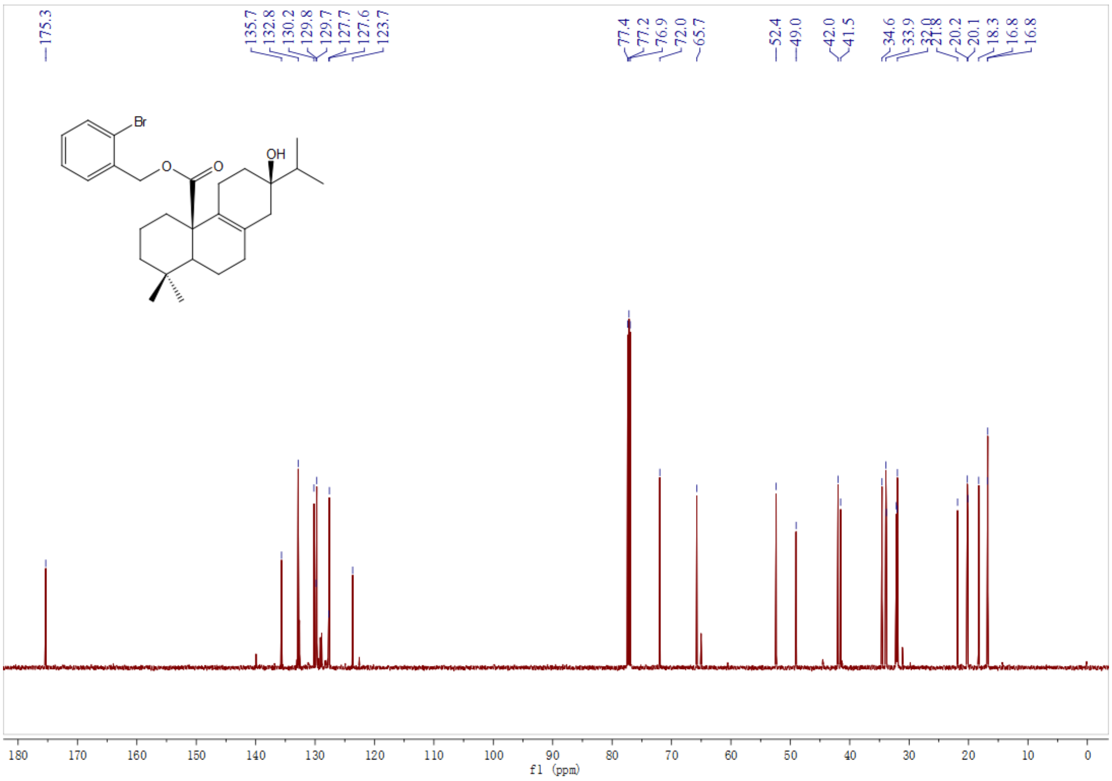

Figure S26. ^{13}C NMR spectrum of the compound **3i**.

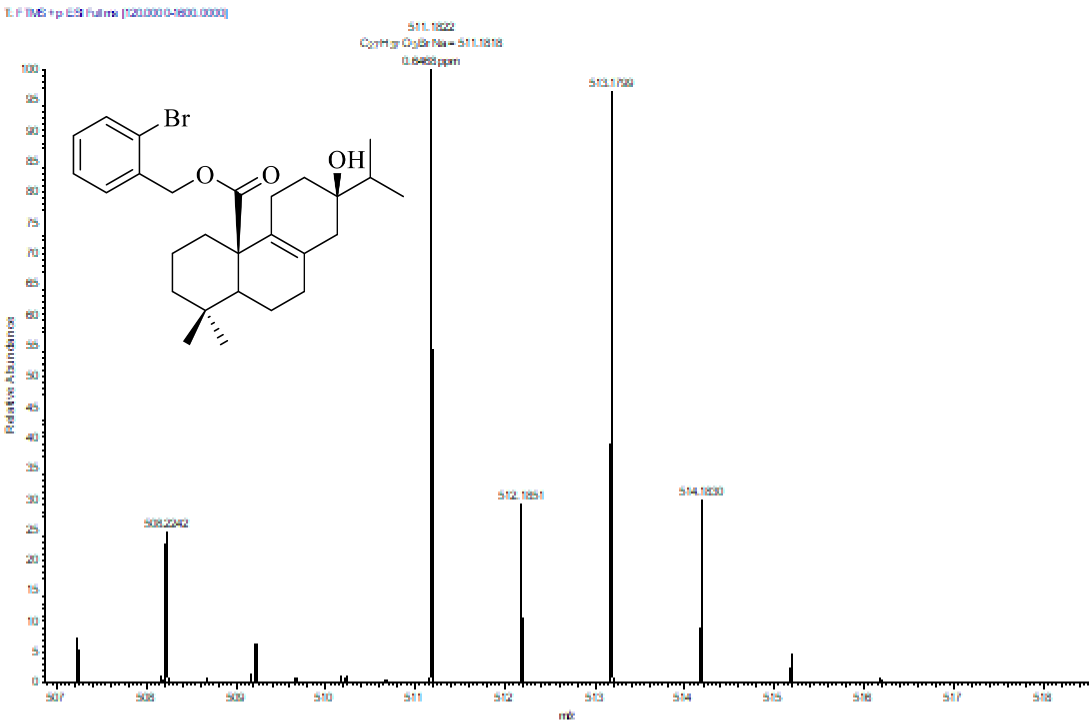

Figure S27. HRMS spectrum of the compound **3i**.

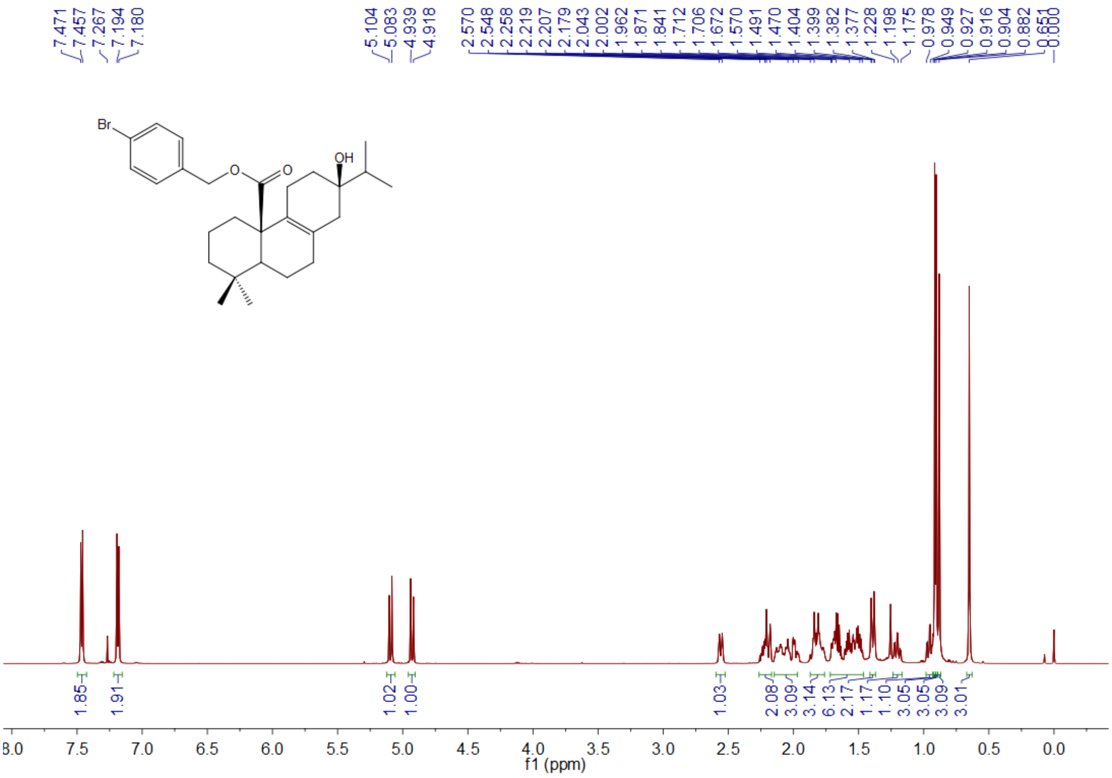

Figure S28. ^{1}H NMR spectrum of the compound **3j**.

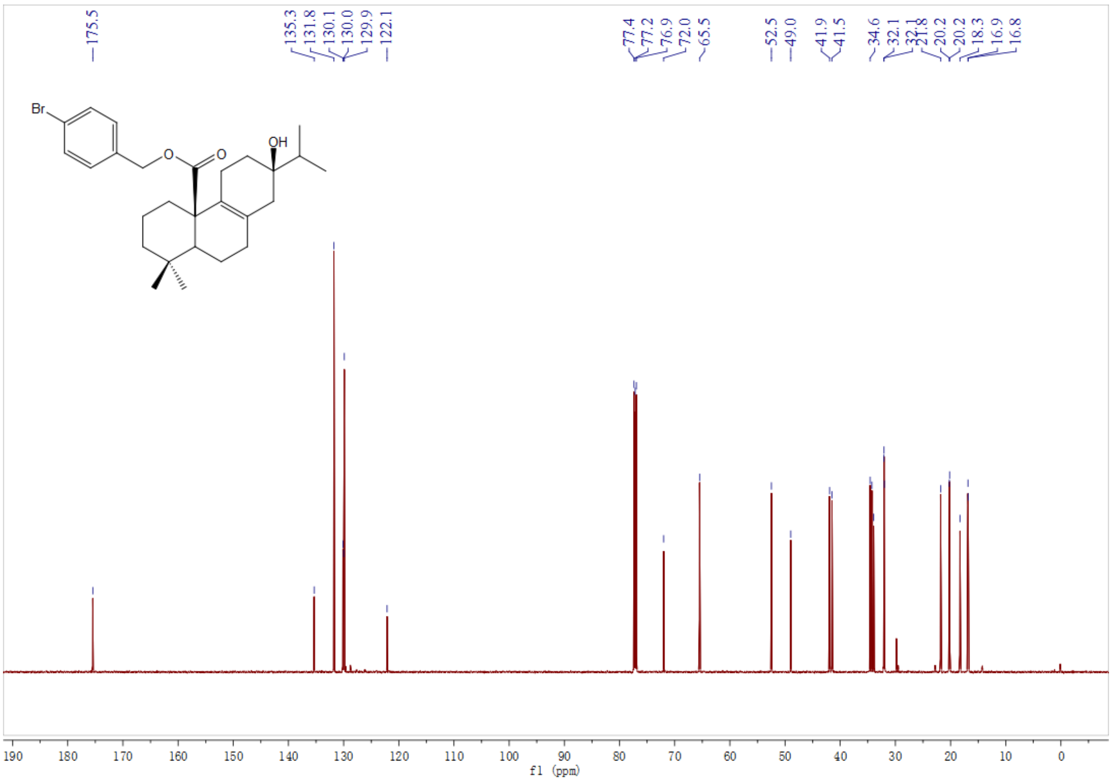

Figure S29. ^{13}C NMR spectrum of the compound **3j**.

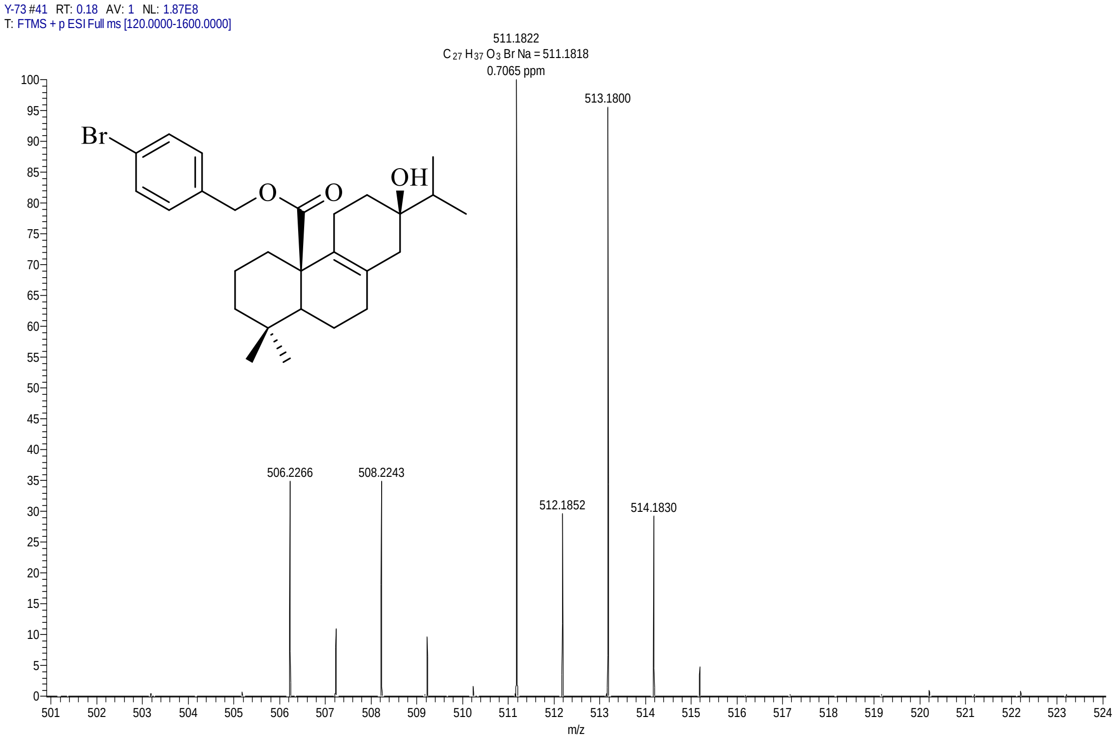

Figure S30. HRMS spectrum of the compound **3j**.

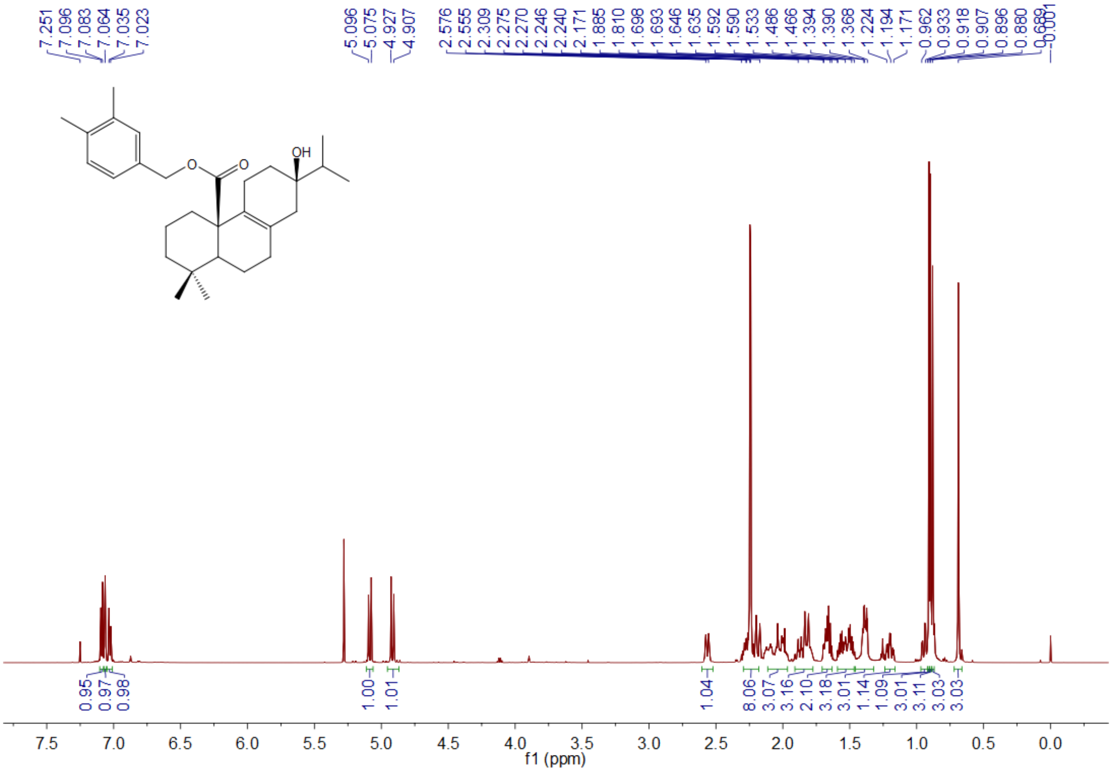

Figure S31. ^{1}H NMR spectrum of the compound **3k**.

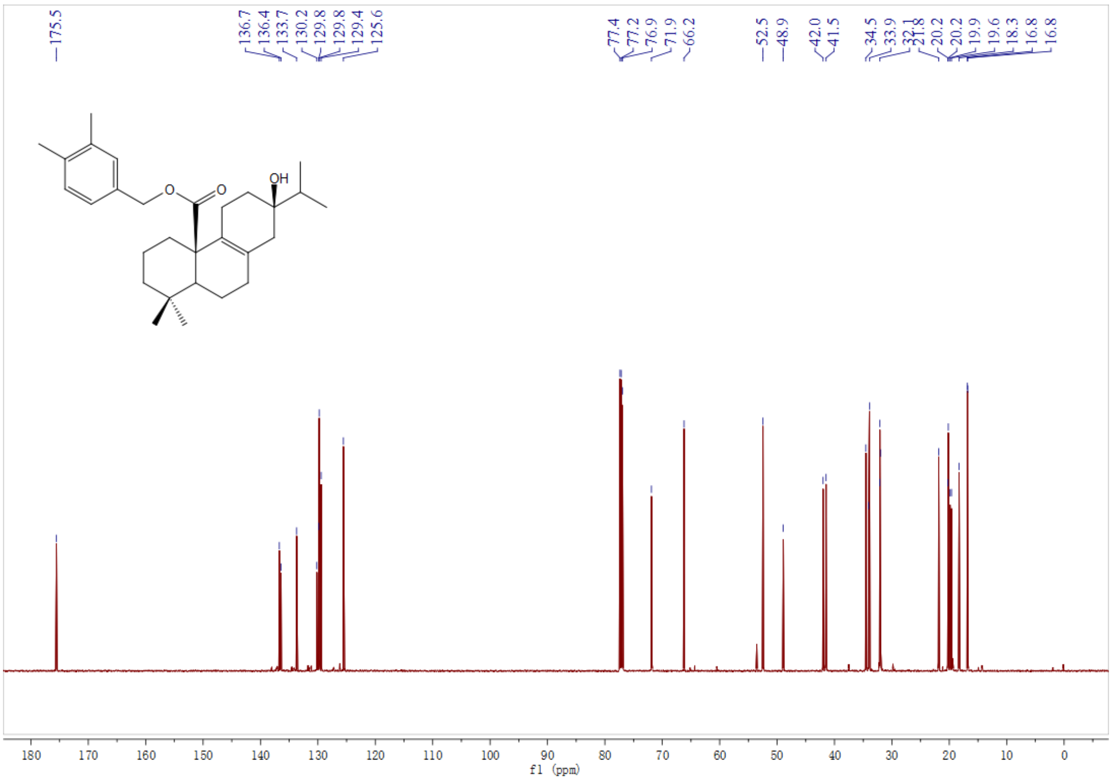

Figure S32. ^{13}C NMR spectrum of the compound **3k**.

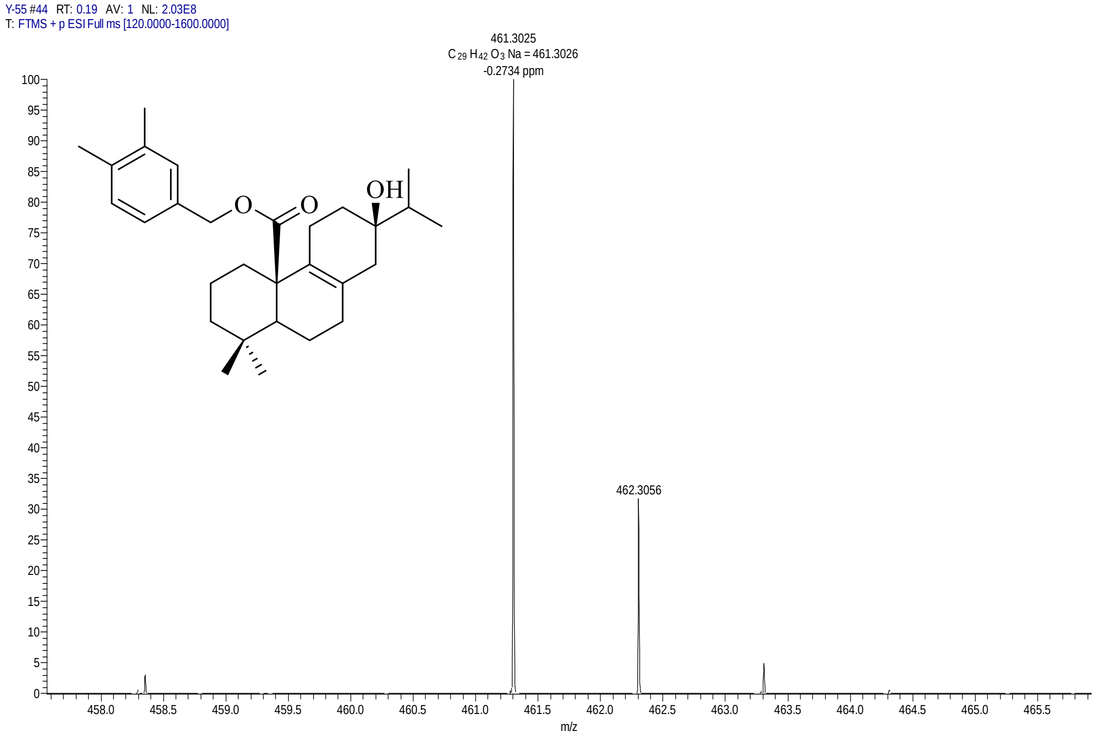

Figure S33. HRMS spectrum of the compound **3k**.

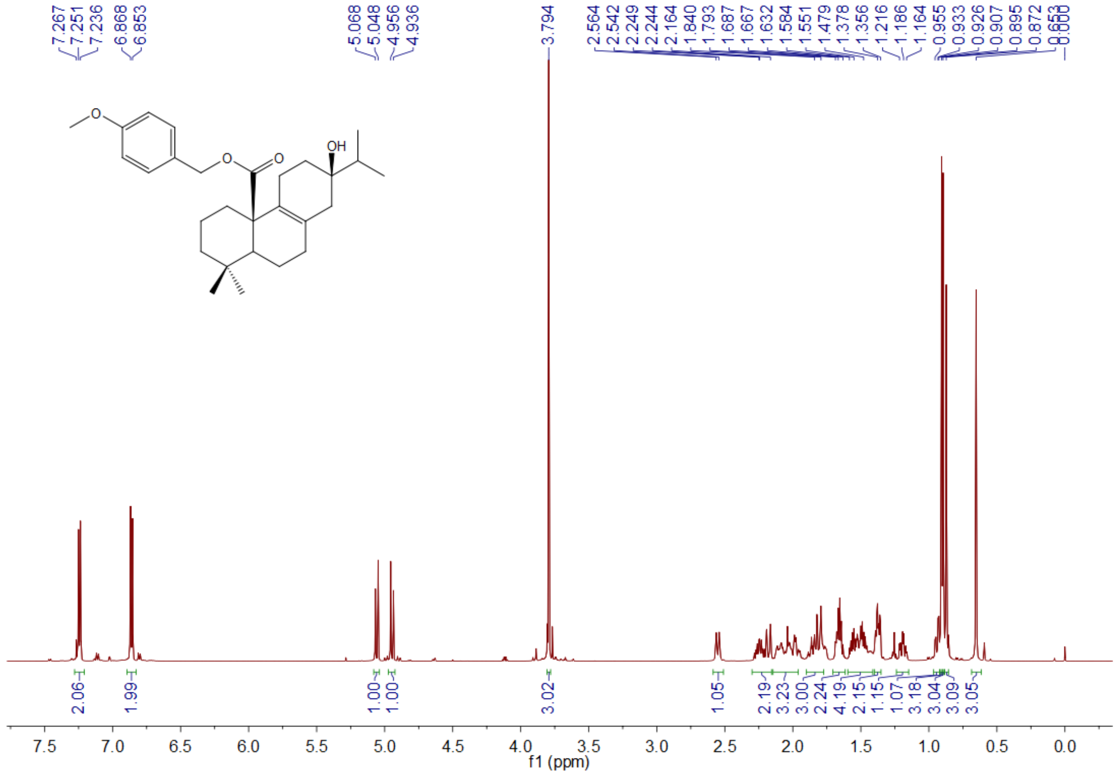

Figure S34. ^{1}H NMR spectrum of the compound **3l**.

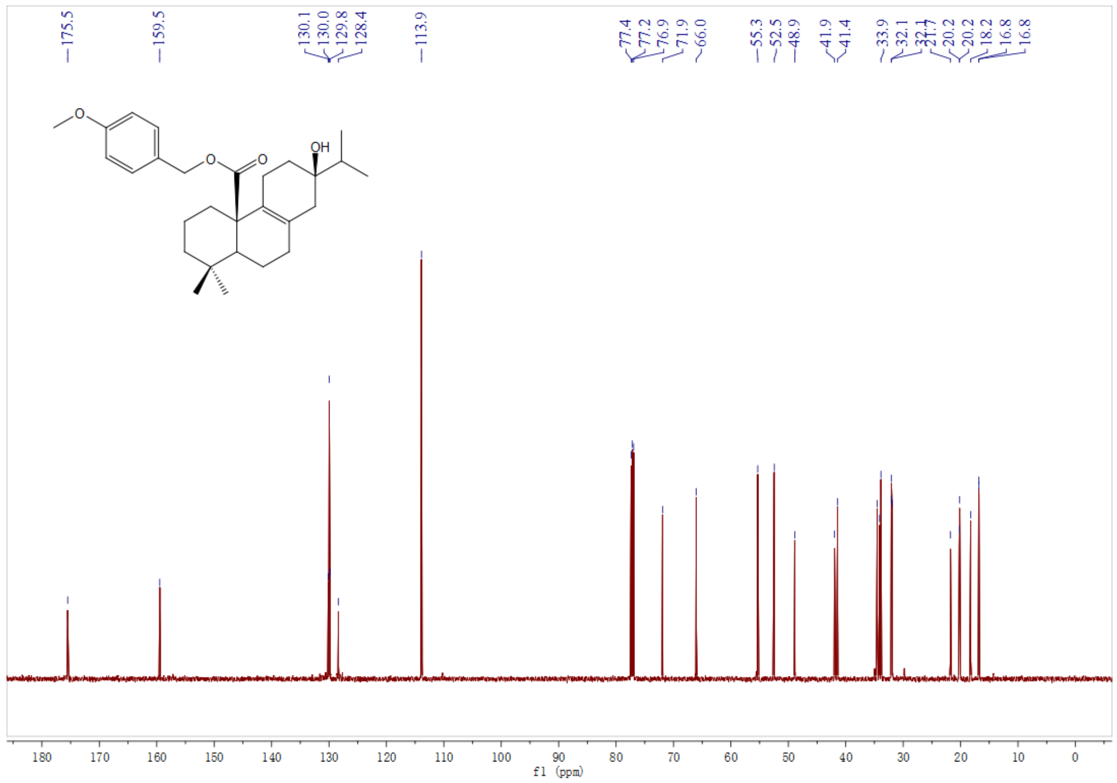

Figure S35. ^{13}C NMR spectrum of the compound **3l**.

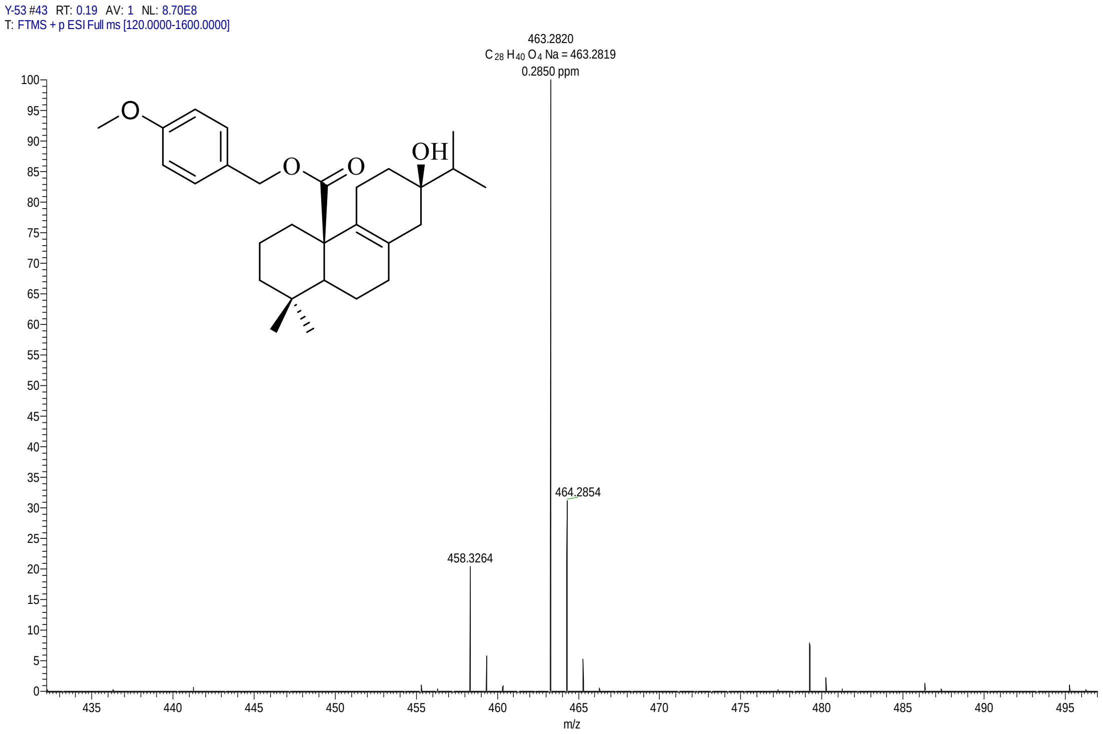

Figure S36. HRMS spectrum of the compound **3l**.

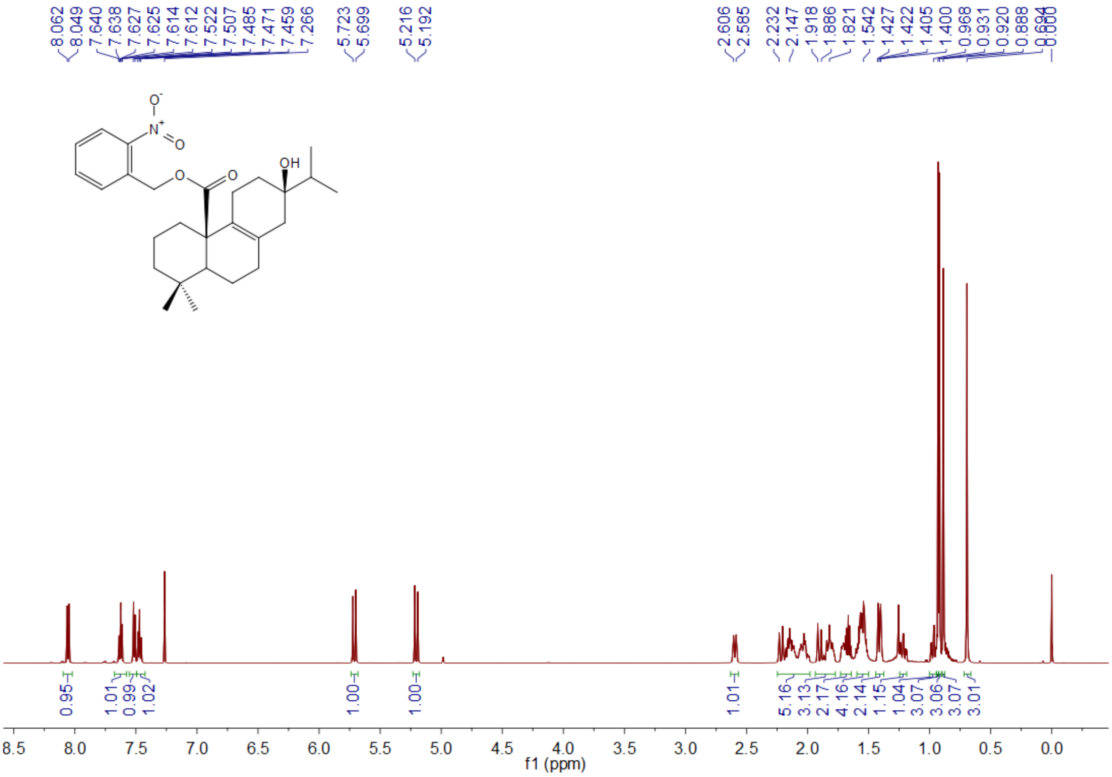

Figure S37. ^{1}H NMR spectrum of the compound **3m**.

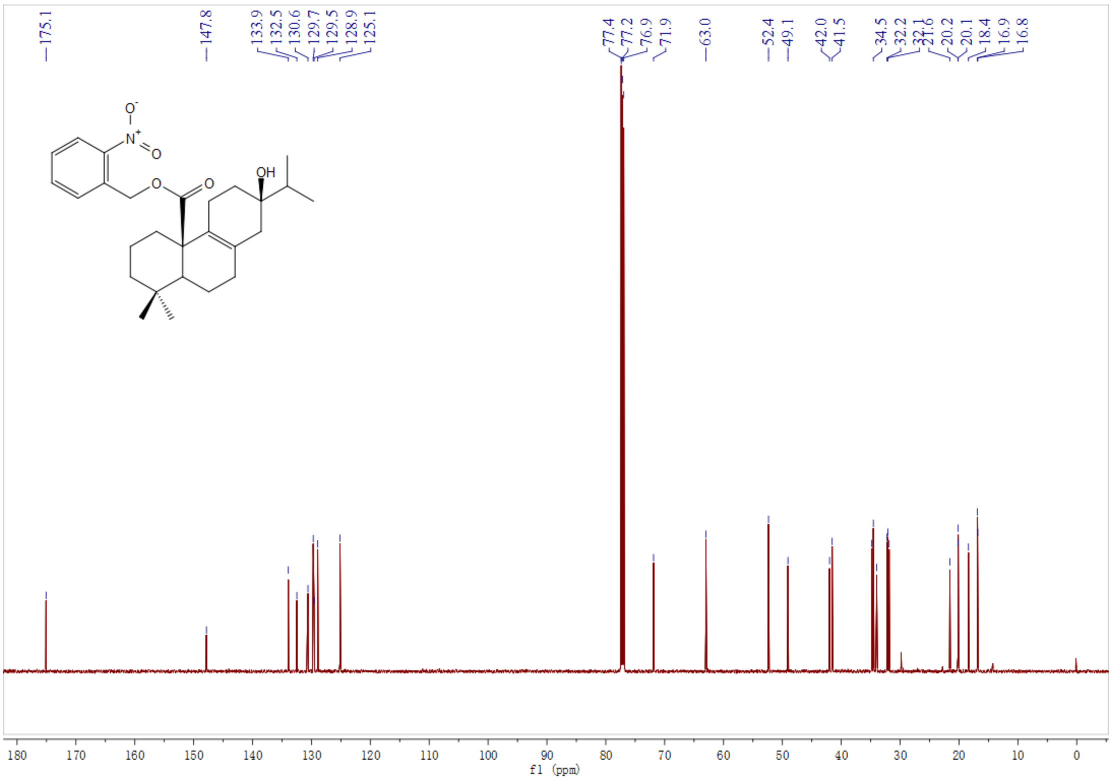

Figure S38. ^{13}C NMR spectrum of the compound **3m**.

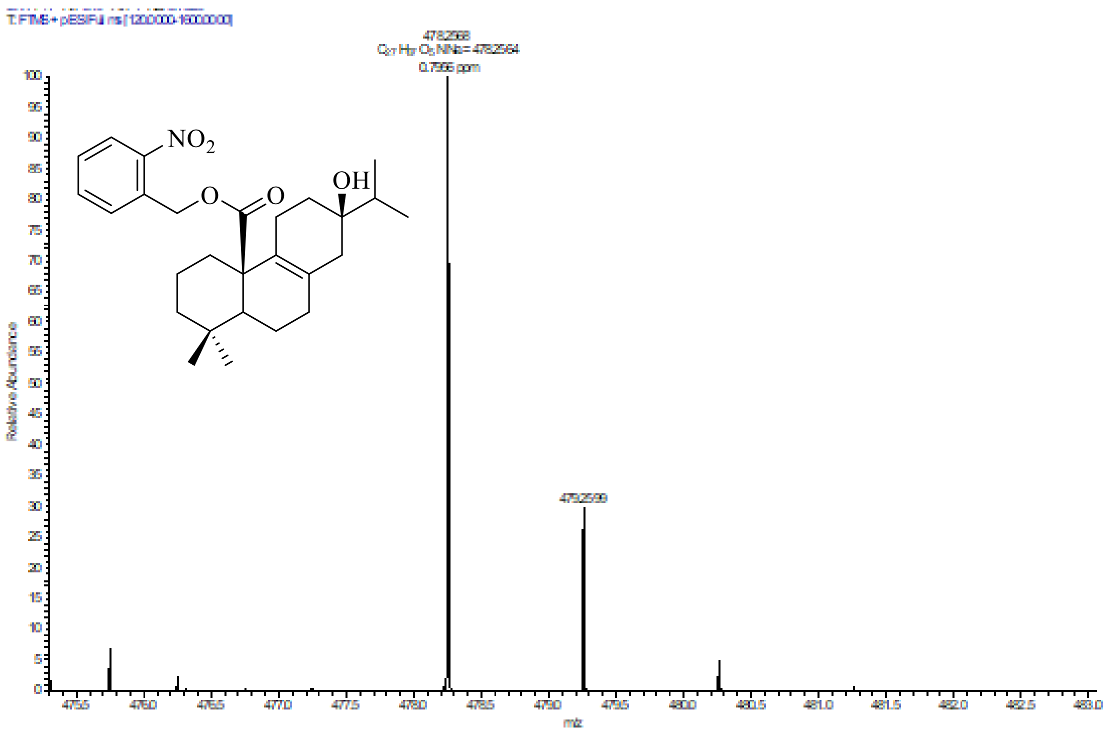

Figure S39. HRMS spectrum of the compound **3m**.

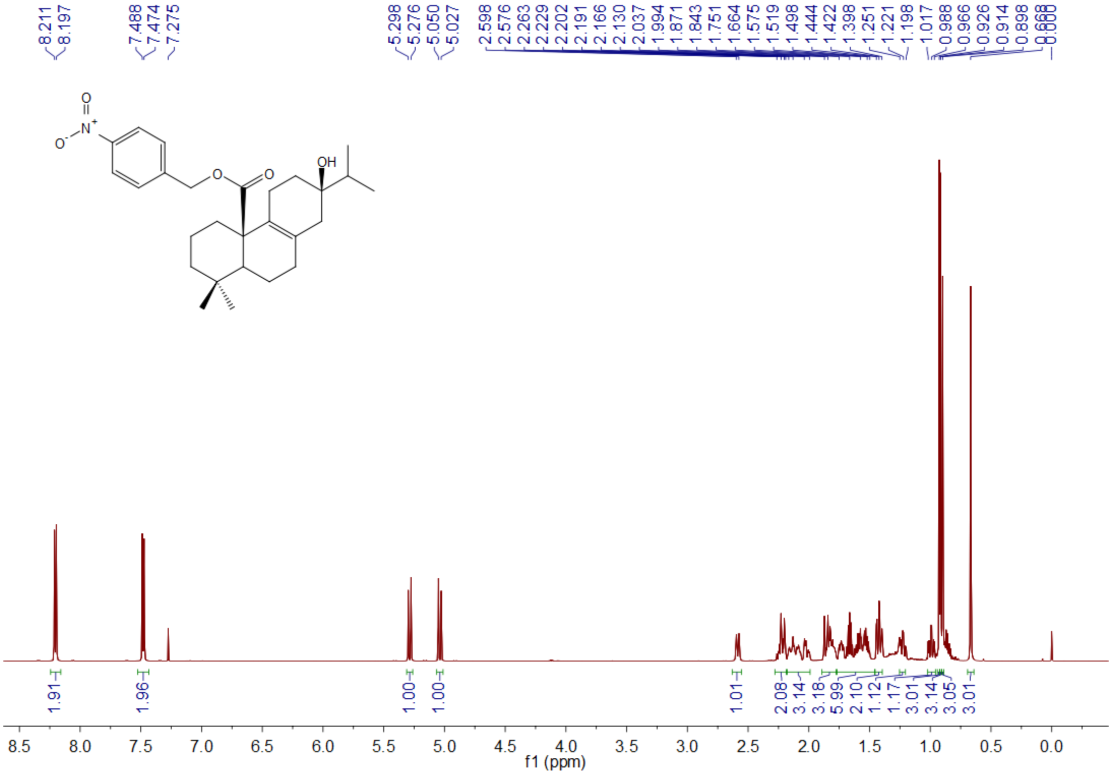

Figure S40. ^{1}H NMR spectrum of the compound **3n**.

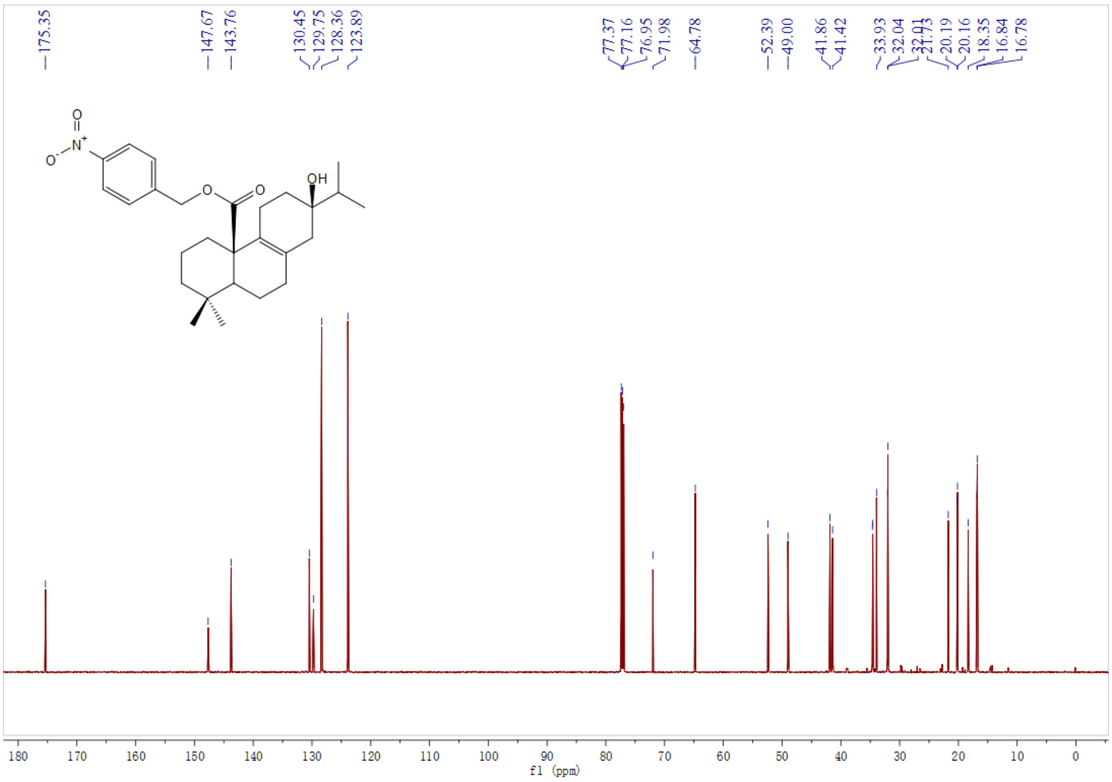

Figure S41. ^{13}C NMR spectrum of the compound **3n**.

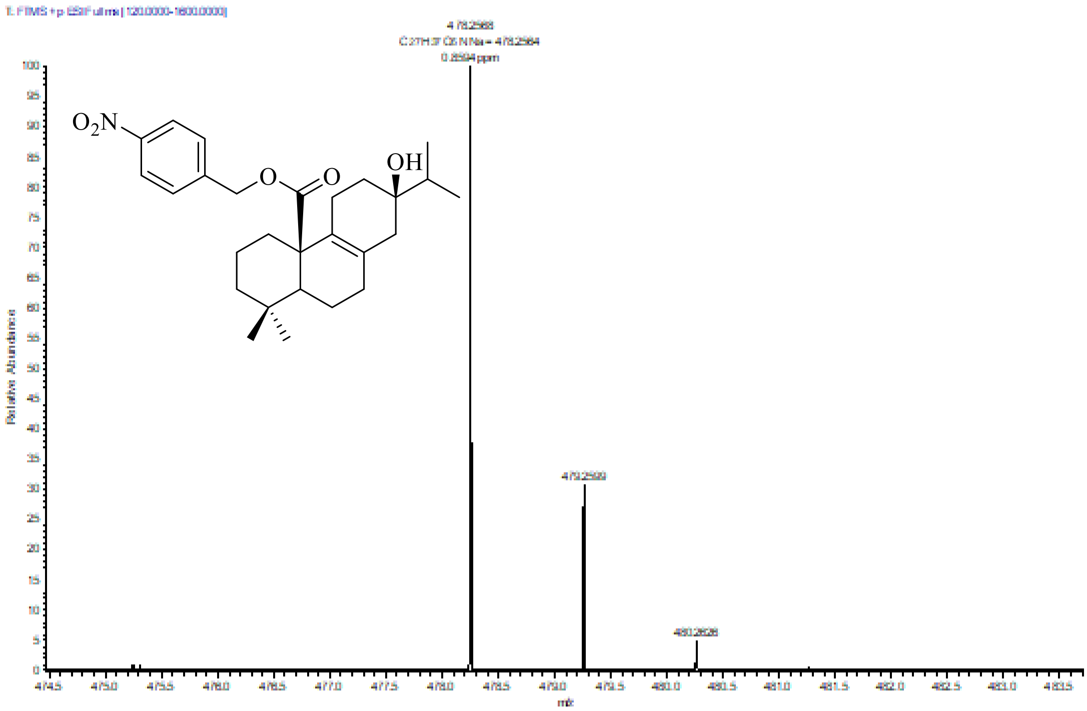

Figure S42. HRMS spectrum of the compound **3n**.

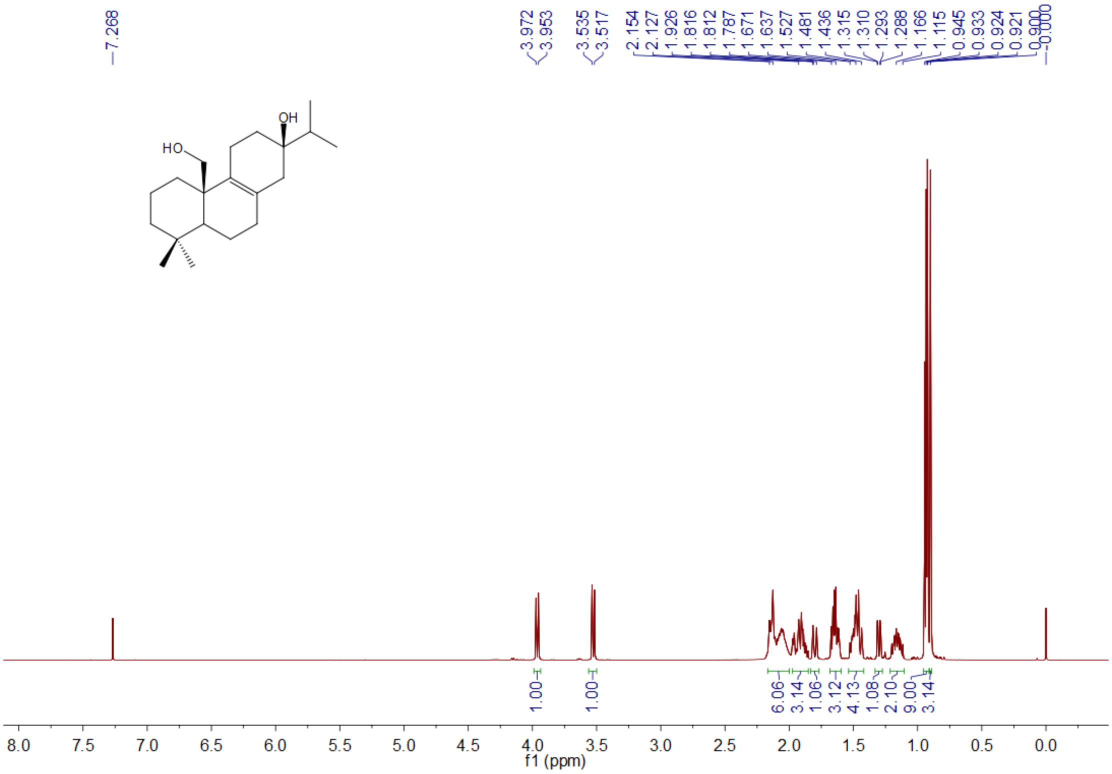

Figure S43. ^{1}H NMR spectrum of the compound **4**.

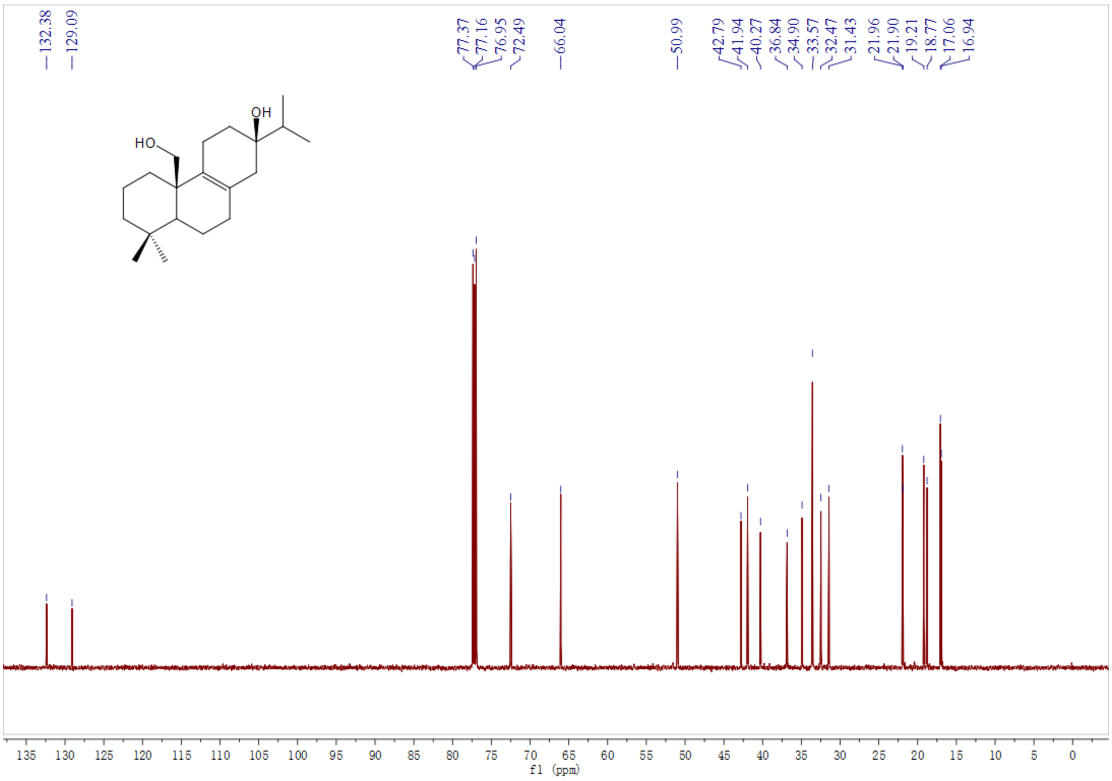

Figure S44. ^{13}C NMR spectrum of the compound **4**.

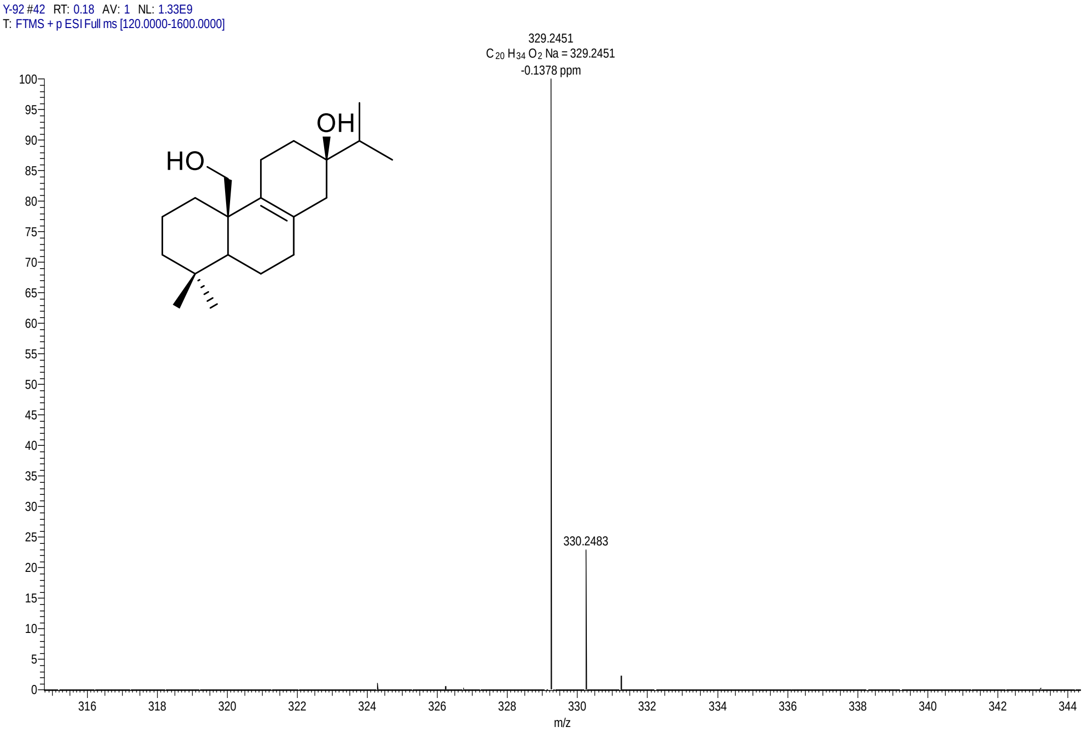

Figure S45. HRMS spectrum of the compound **4**.

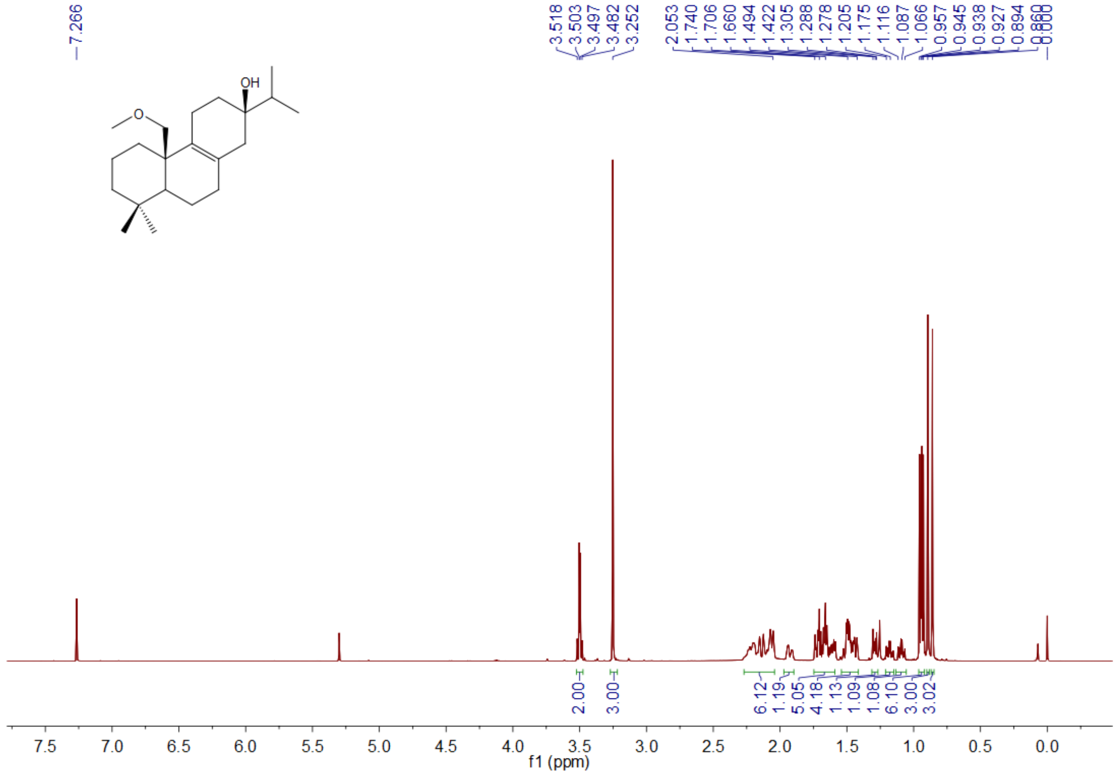

Figure S46. ^{1}H NMR spectrum of the compound **5a**.

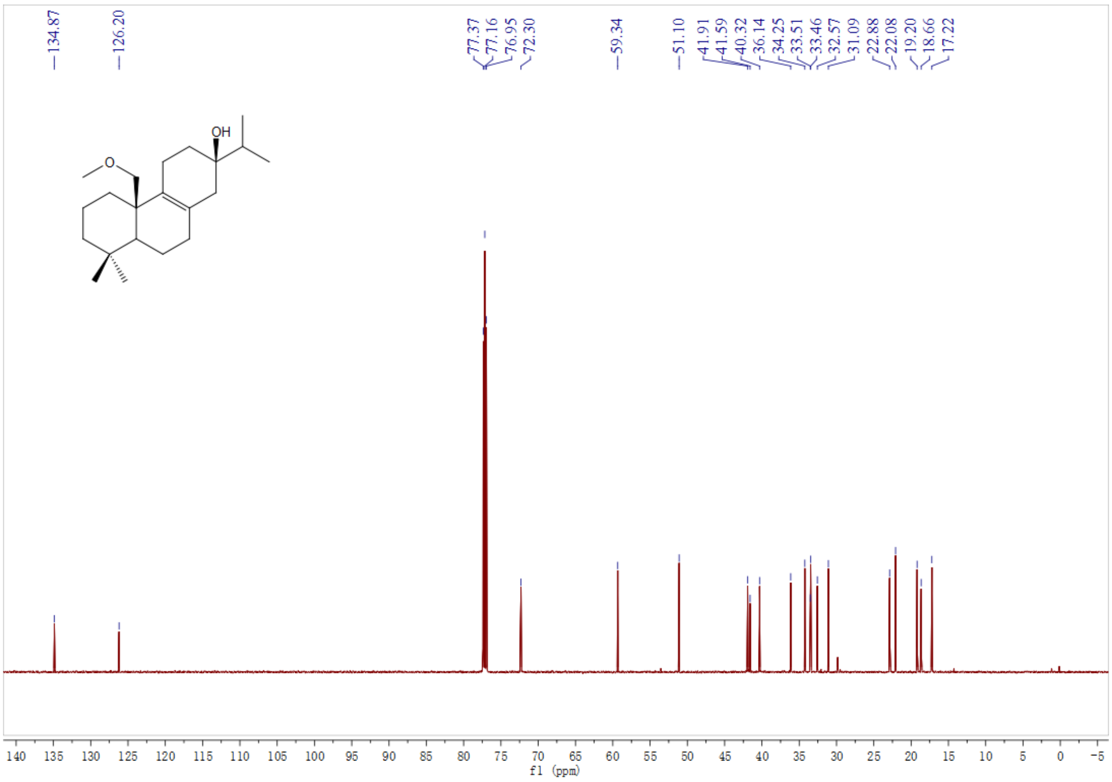

Figure S47. ^{13}C NMR spectrum of the compound **5a**.

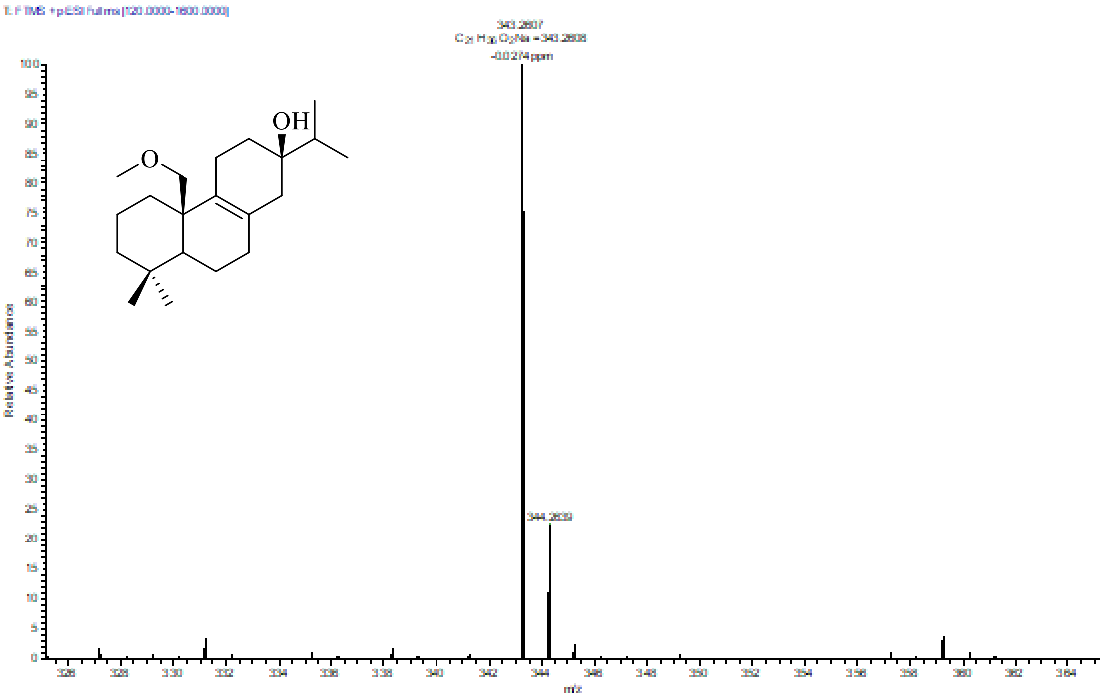

Figure S48. HRMS spectrum of the compound **5a**.

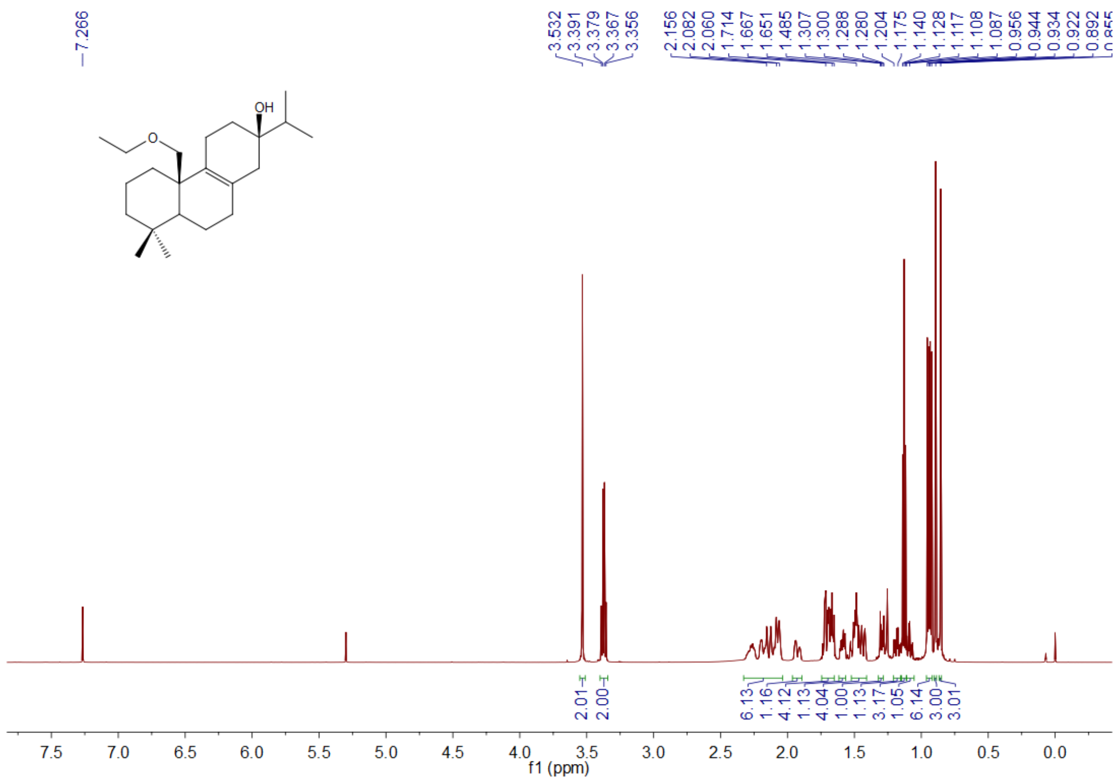

Figure S49. ^{1}H NMR spectrum of the compound **5b**.

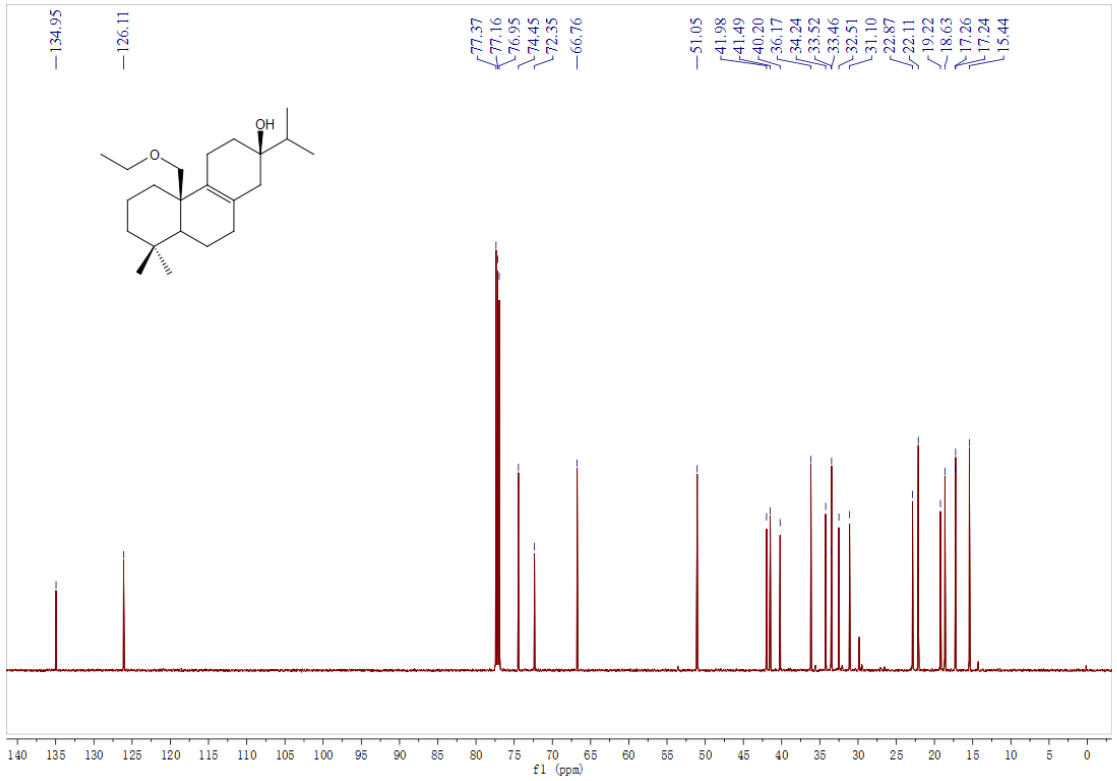

Figure S50. ^{13}C NMR spectrum of the compound **5b**.

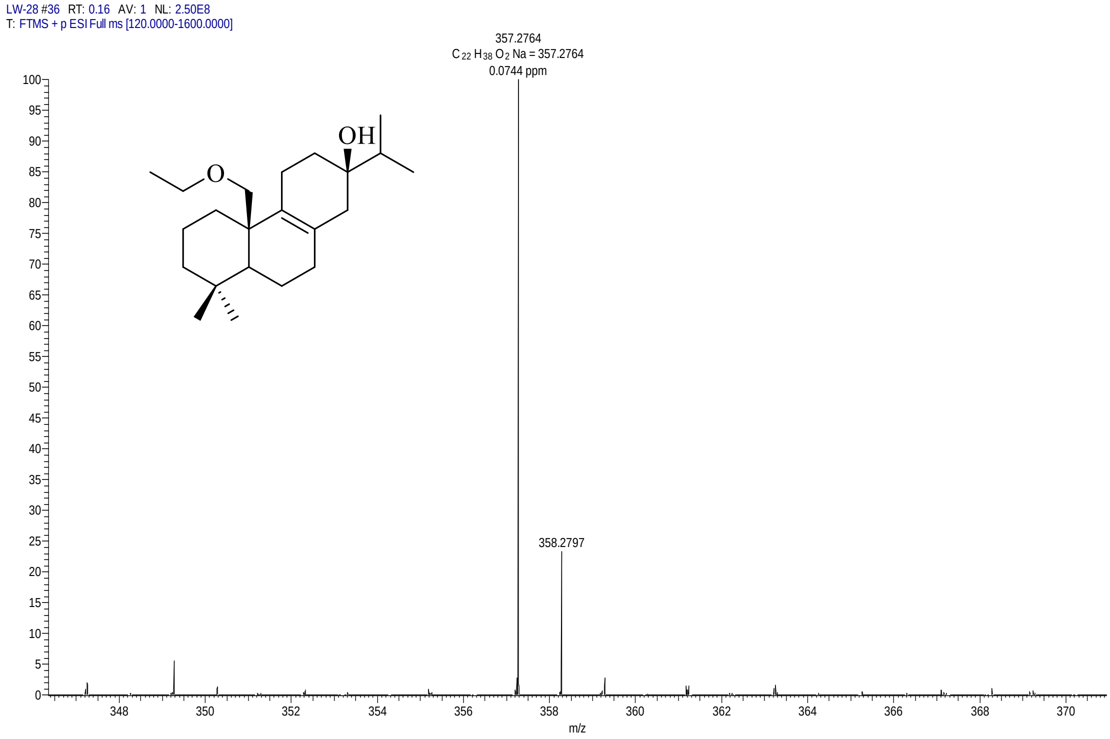

Figure S51. HRMS spectrum of the compound **5b**.

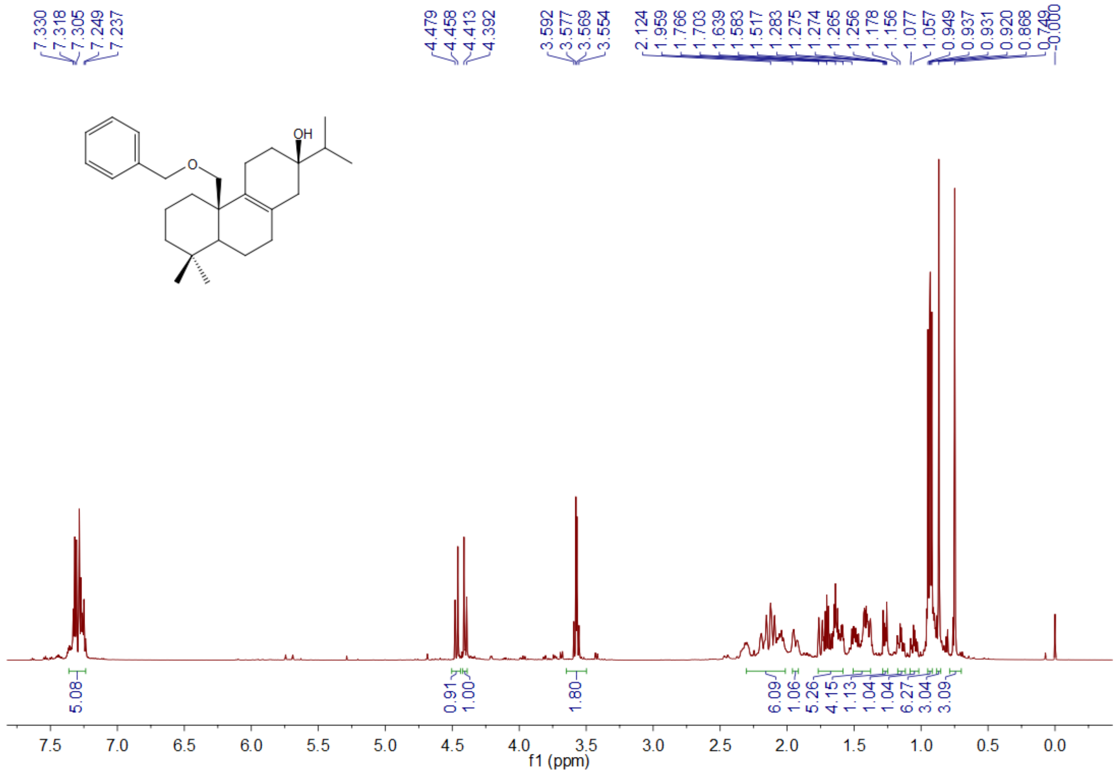

Figure S52. ^{1}H NMR spectrum of the compound **5c**.

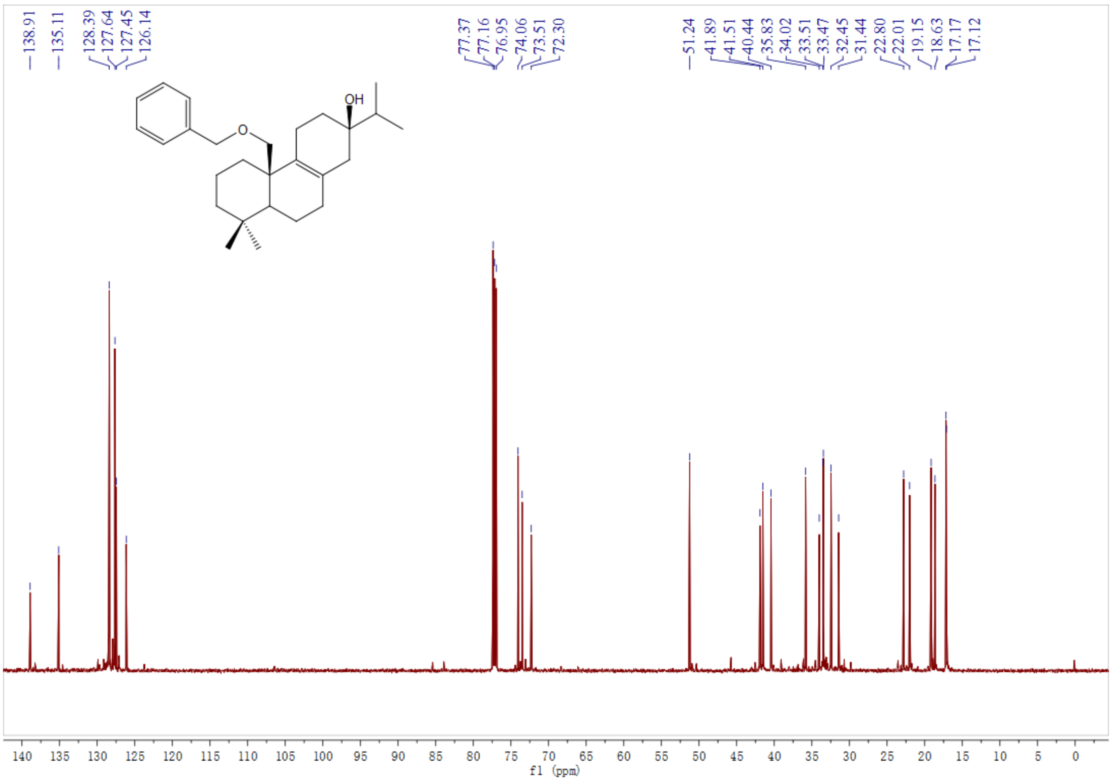

Figure S53. ^{13}C NMR spectrum of the compound **5c**.

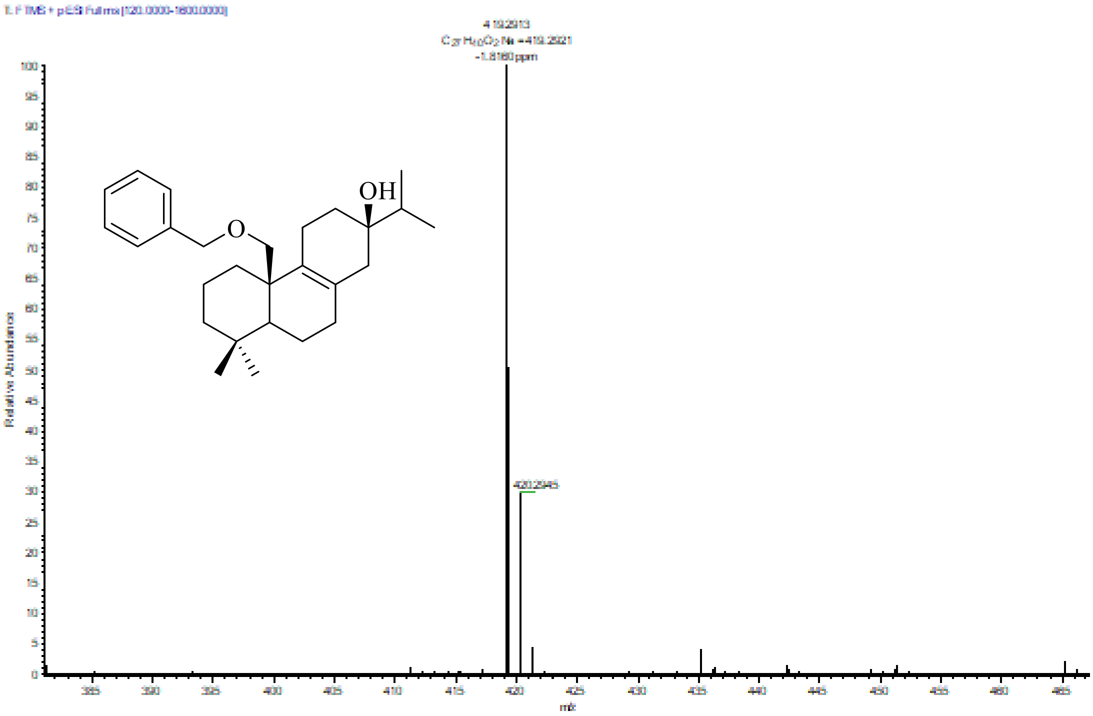

Figure S54. HRMS spectrum of the compound **5c**.

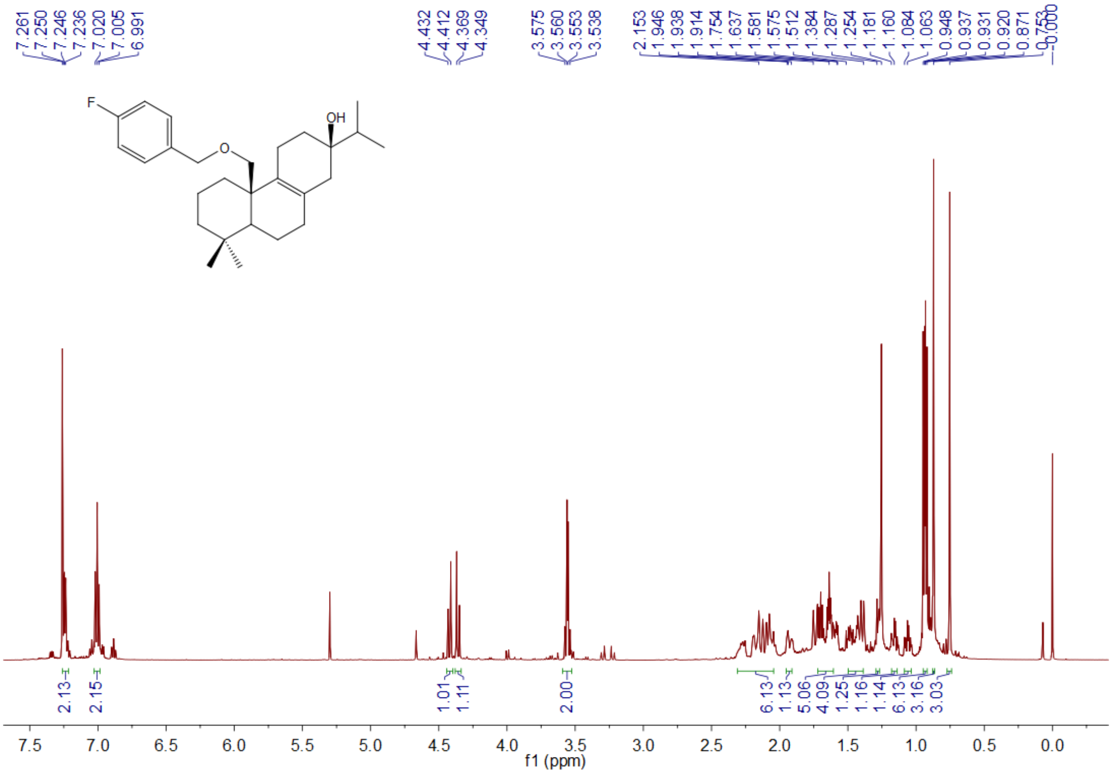

Figure S55. ^{1}H NMR spectrum of the compound **5d**.

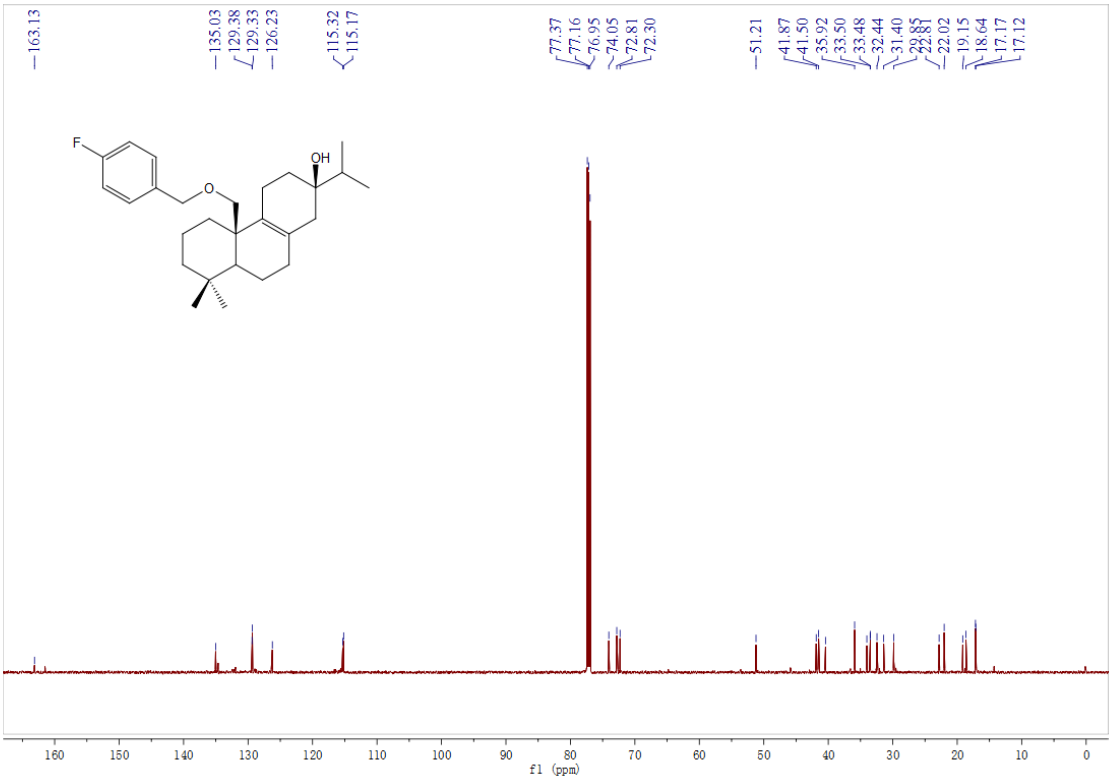

Figure S56. ^{13}C NMR spectrum of the compound **5d**.

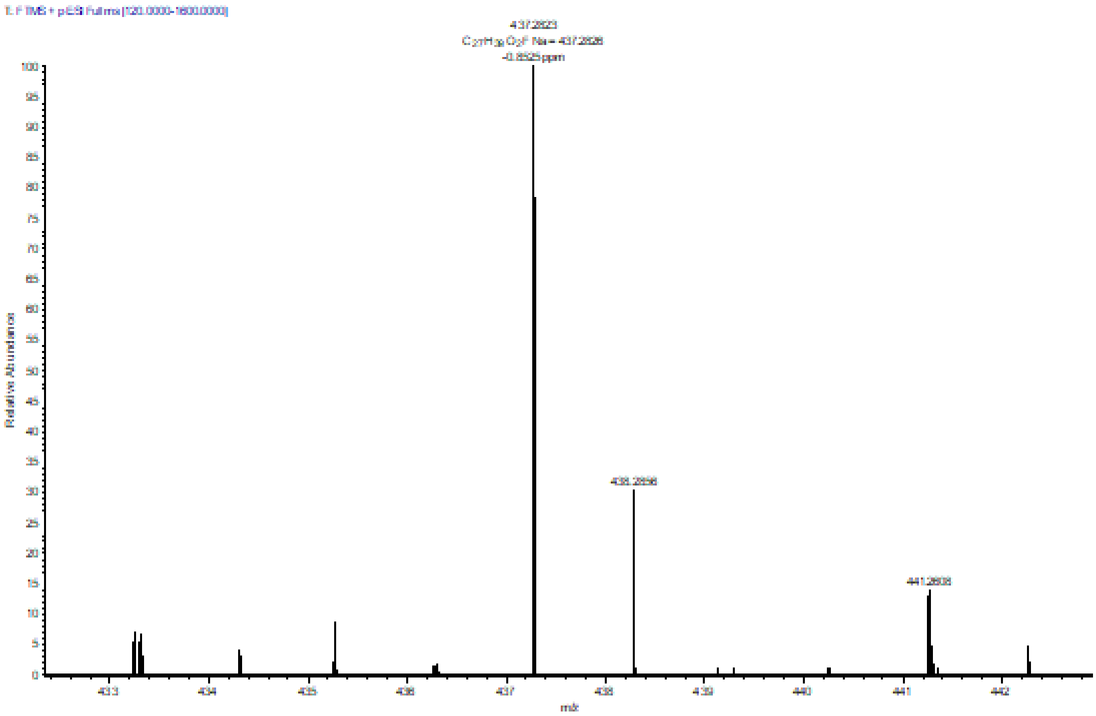

Figure S57. HRMS spectrum of the compound **5d**.

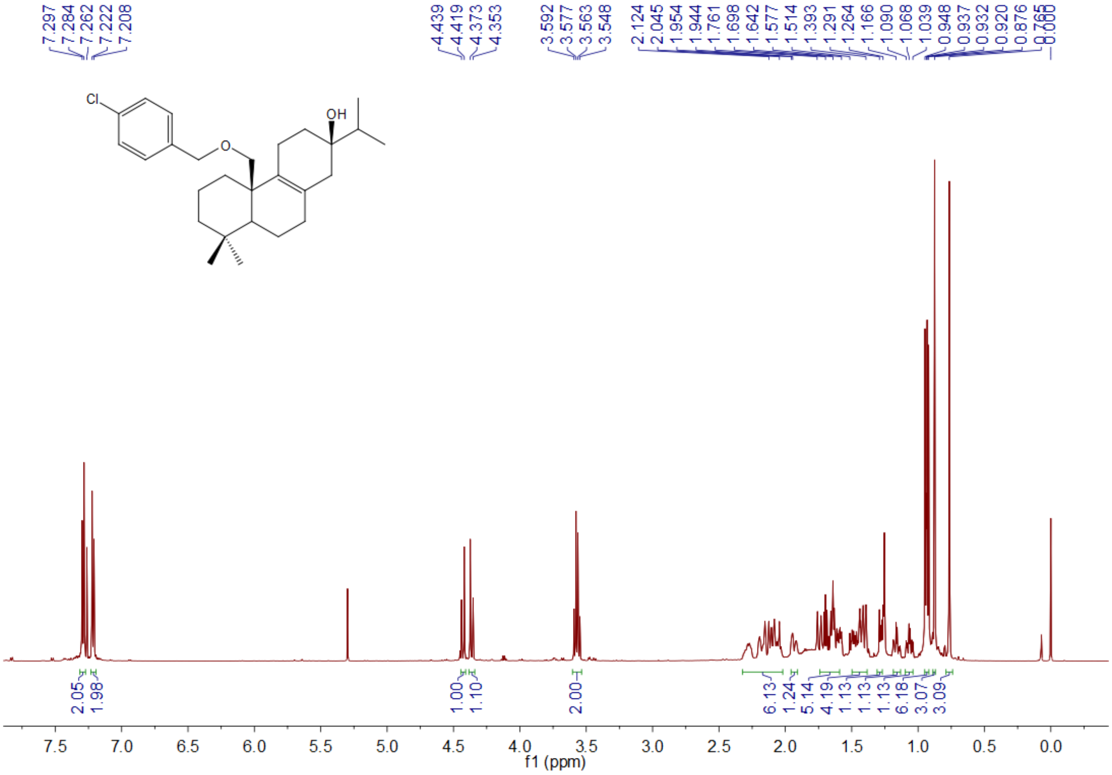

Figure S58. ^{1}H NMR spectrum of the compound **5e**.

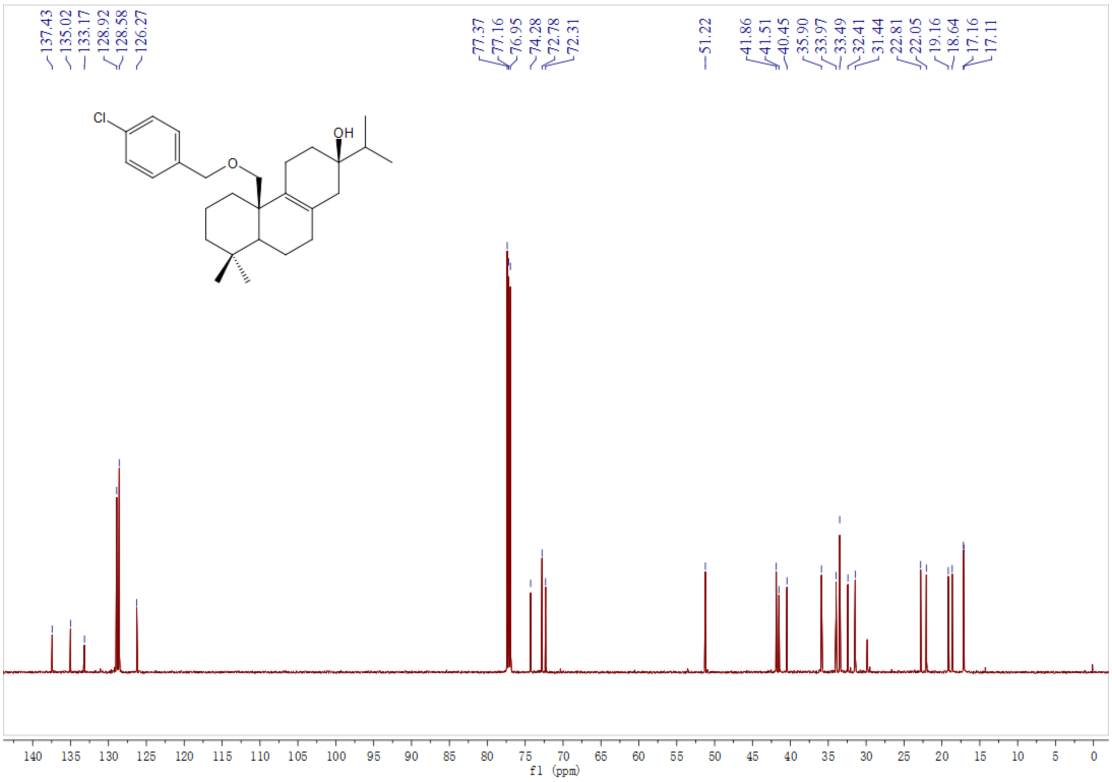

Figure S59. ^{13}C NMR spectrum of the compound **5e**.

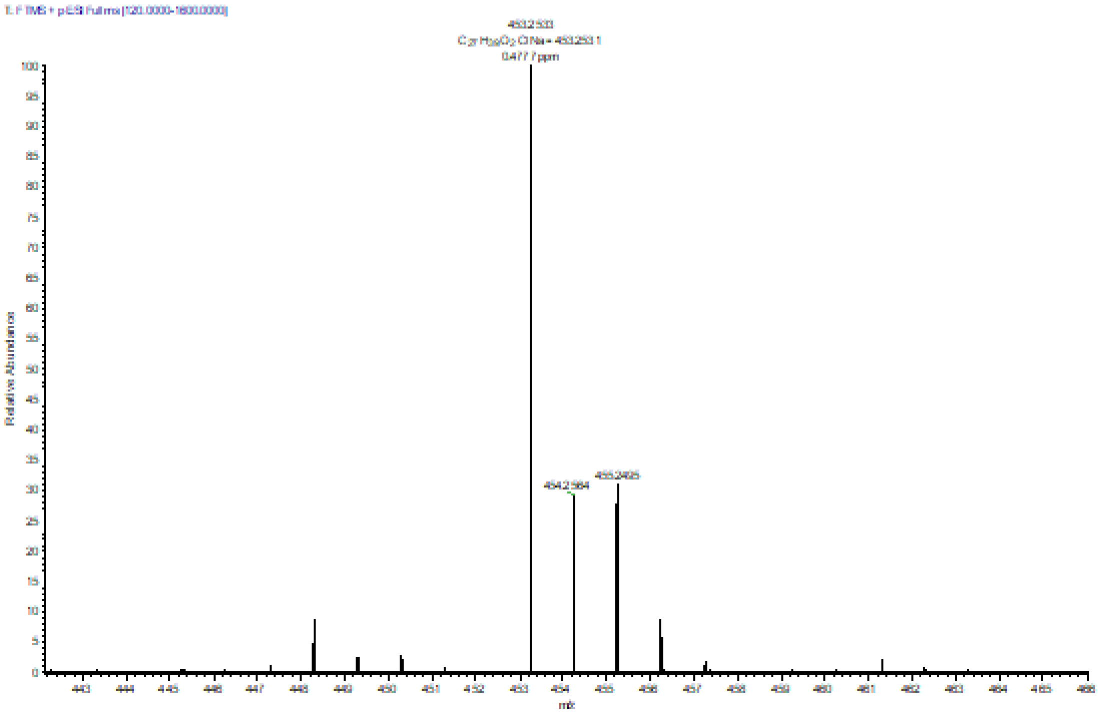

Figure S60. HRMS spectrum of the compound **5e**.

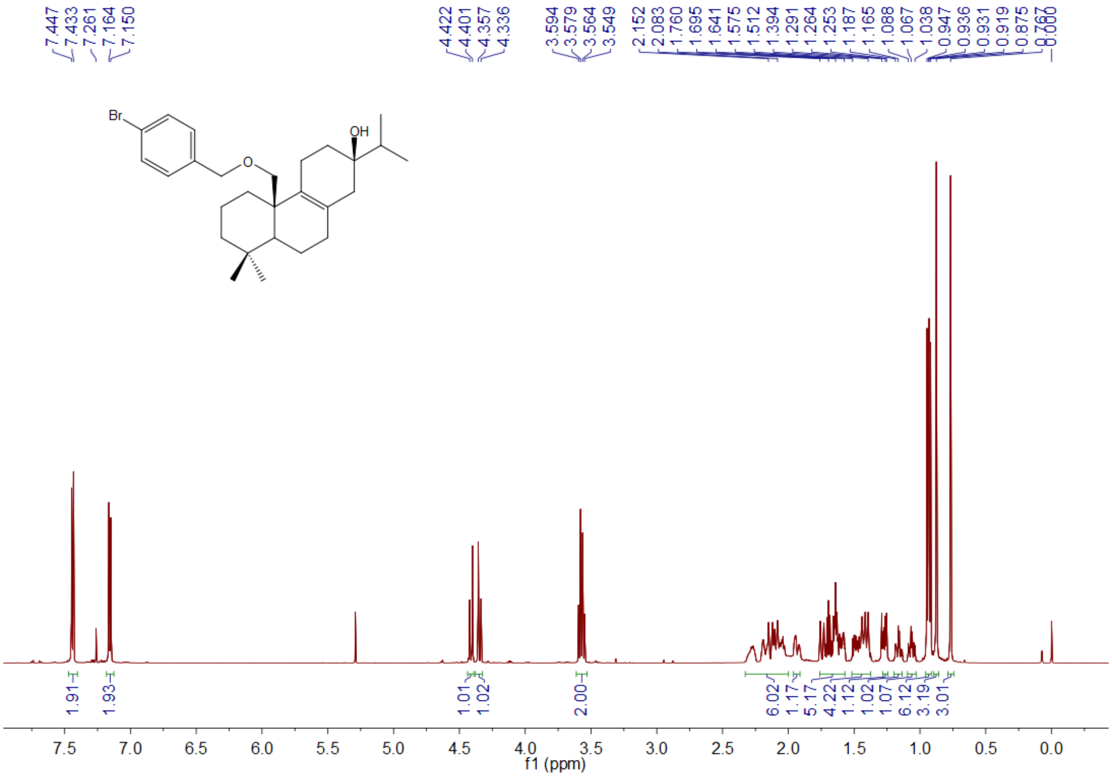

Figure S61. ^{1}H NMR spectrum of the compound **5f**.

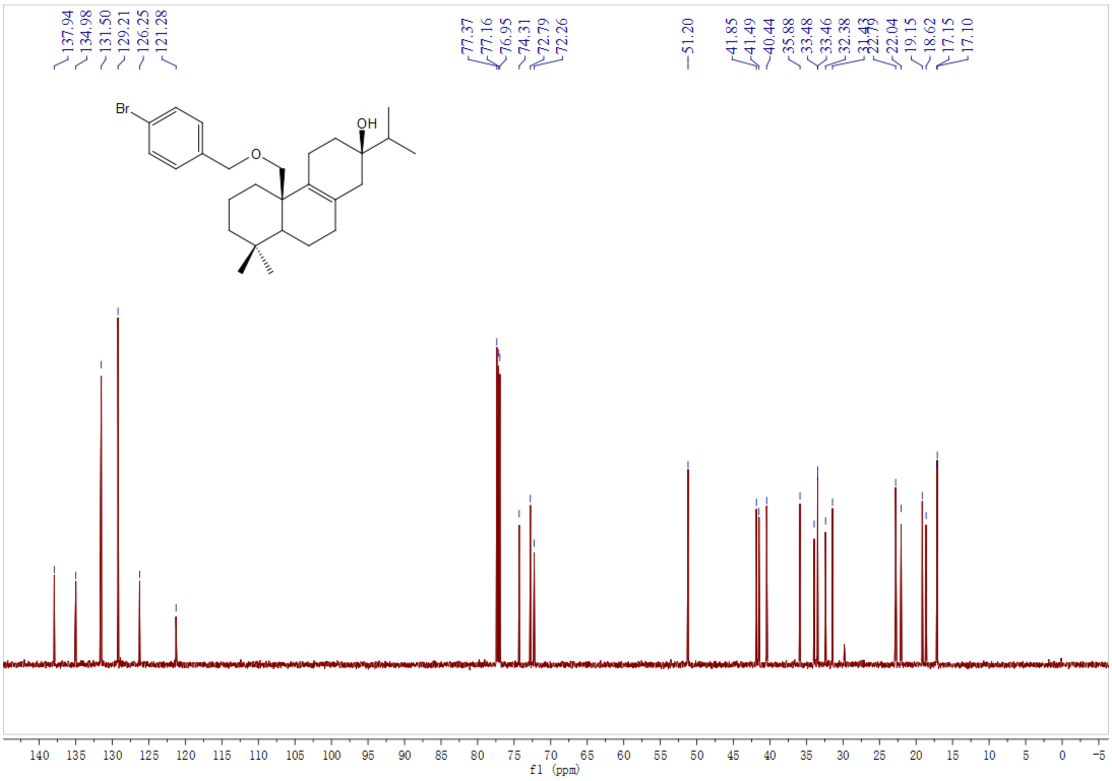

Figure S62. ^{13}C NMR spectrum of the compound **5f**.

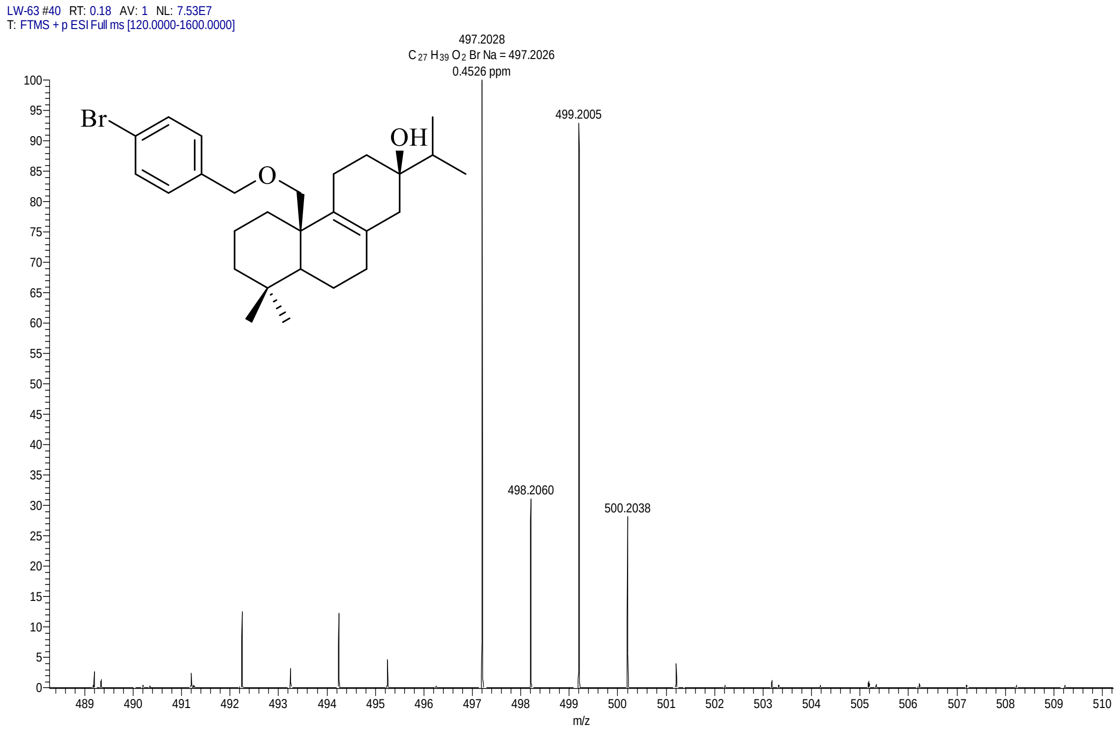

Figure S63. HRMS spectrum of the compound **5f**.

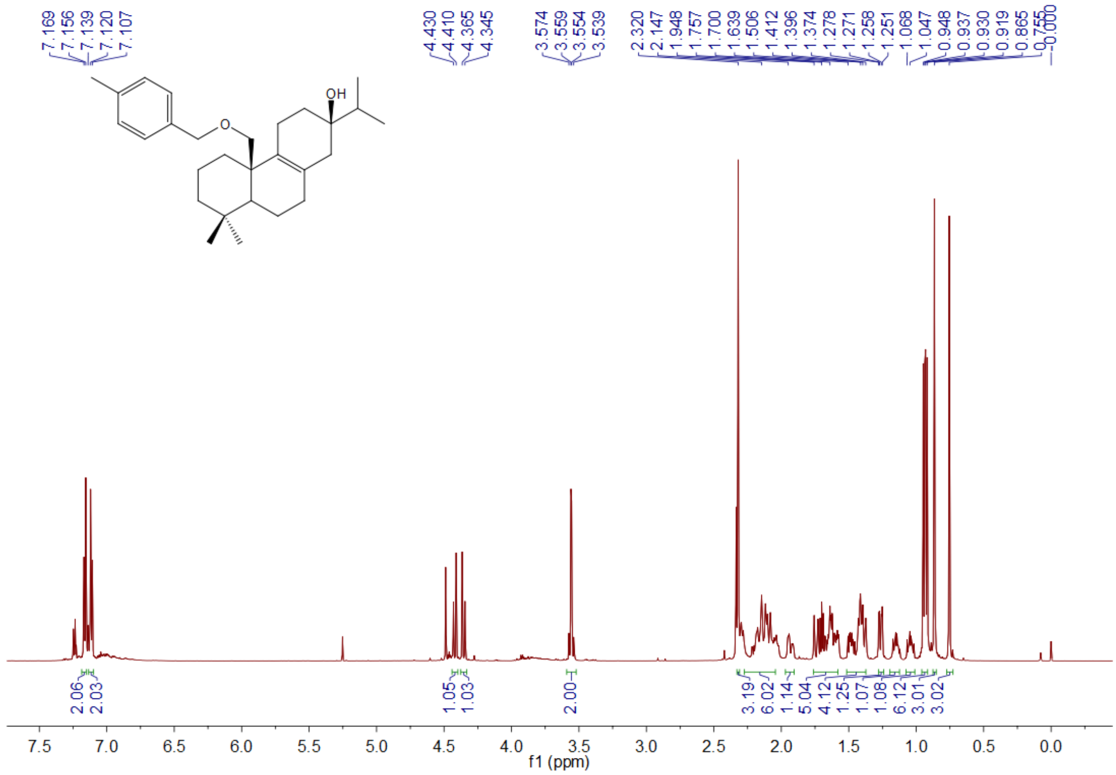

Figure S64. ^{1}H NMR spectrum of the compound **5g**.

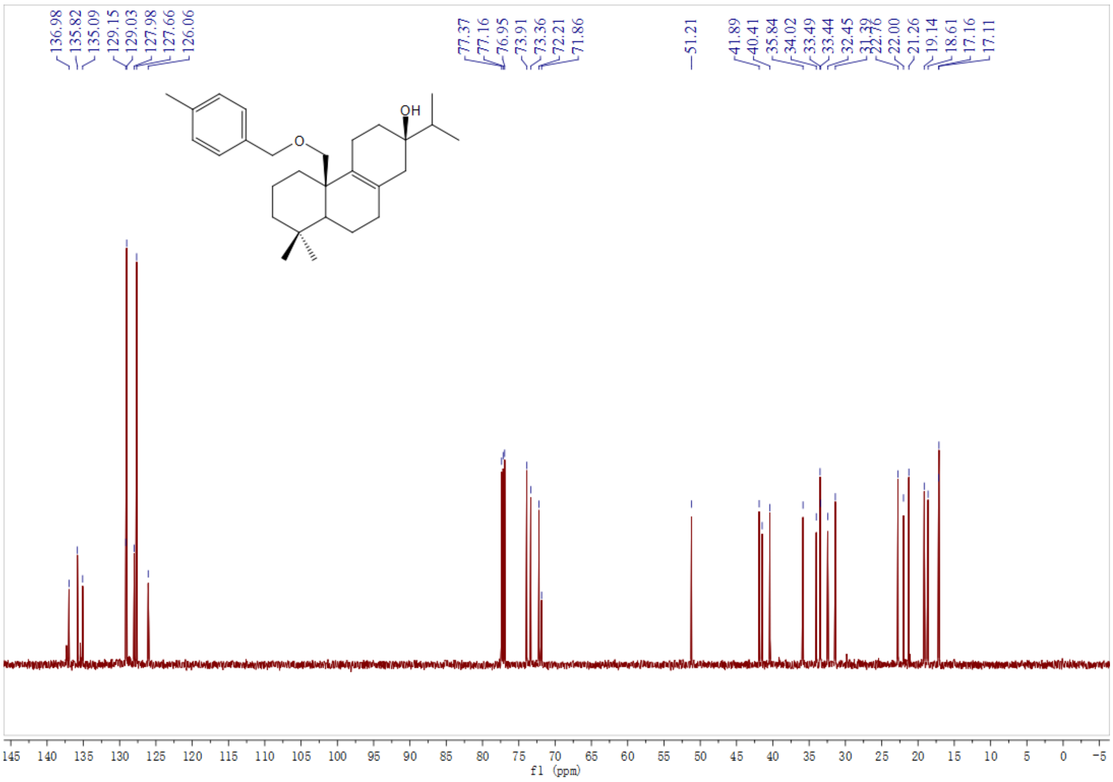

Figure S65. ^{13}C NMR spectrum of the compound **5g**.

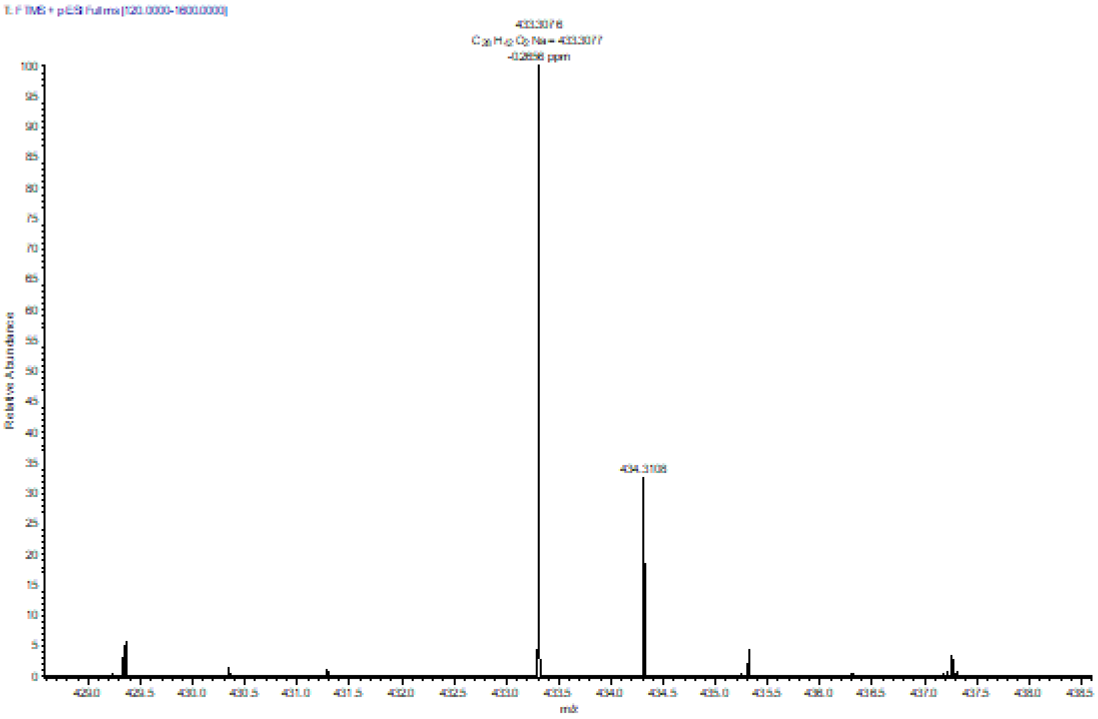

Figure S66. HRMS spectrum of the compound **5g**.

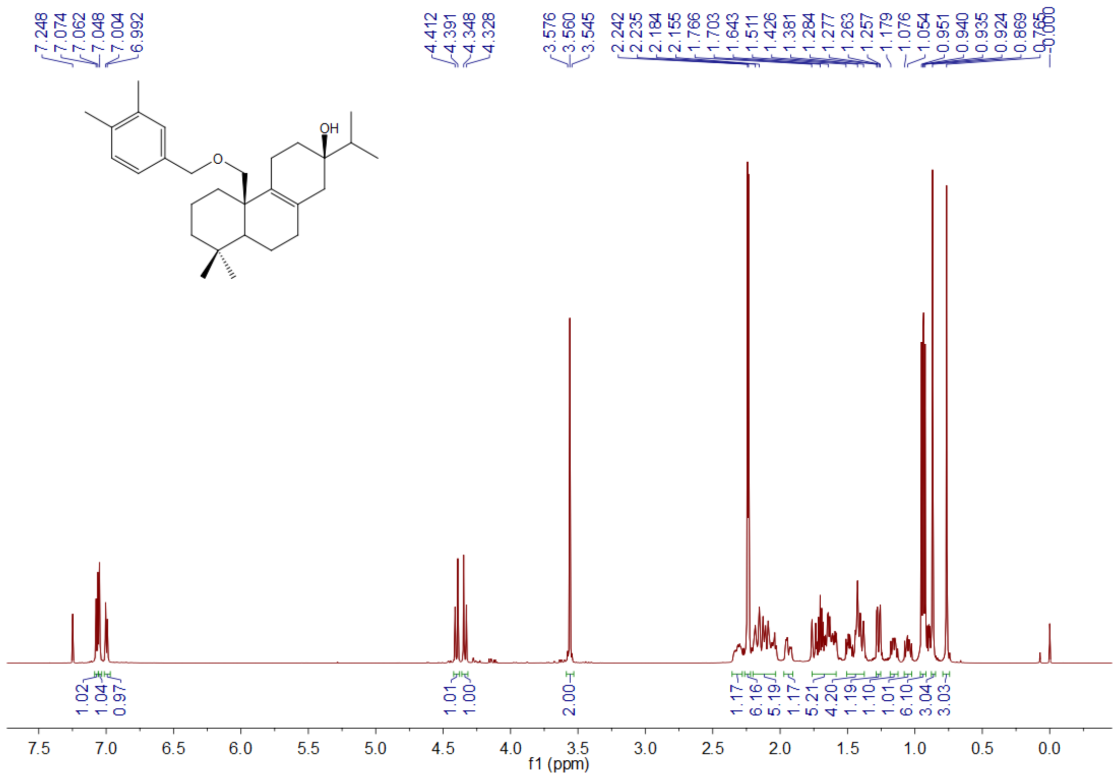

Figure S67. ¹H NMR spectrum of the compound **5h**.

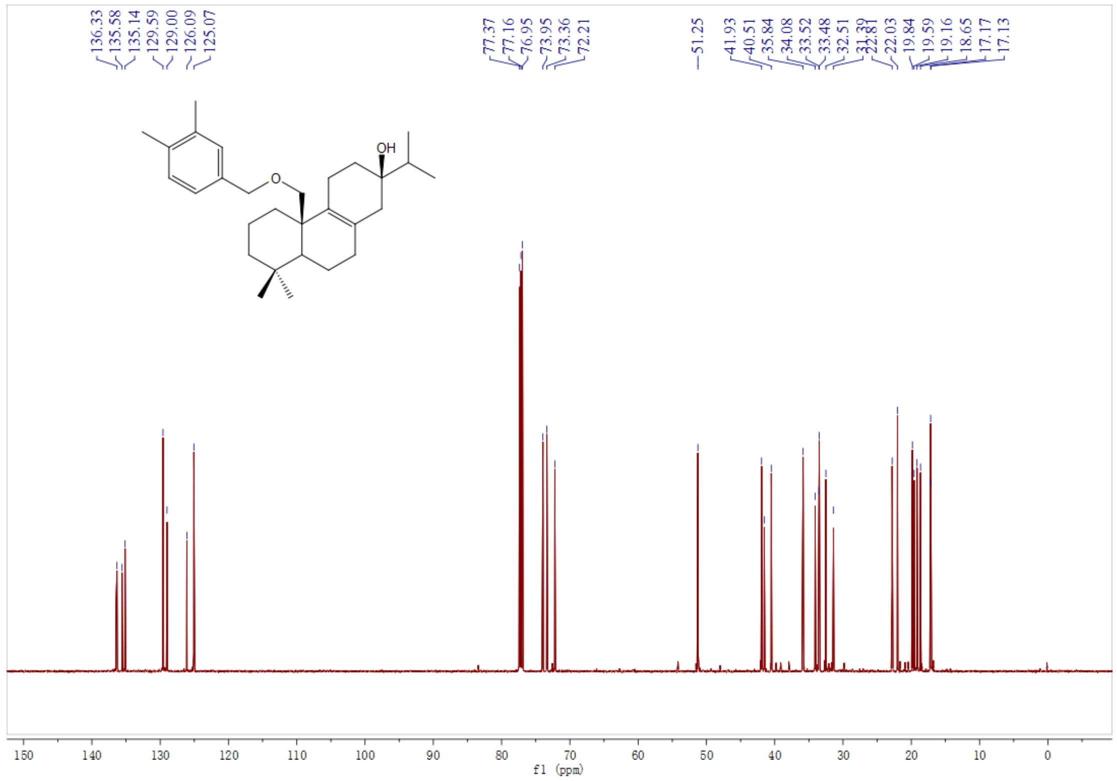

Figure S68. ¹³C NMR spectrum of the compound **5h**.

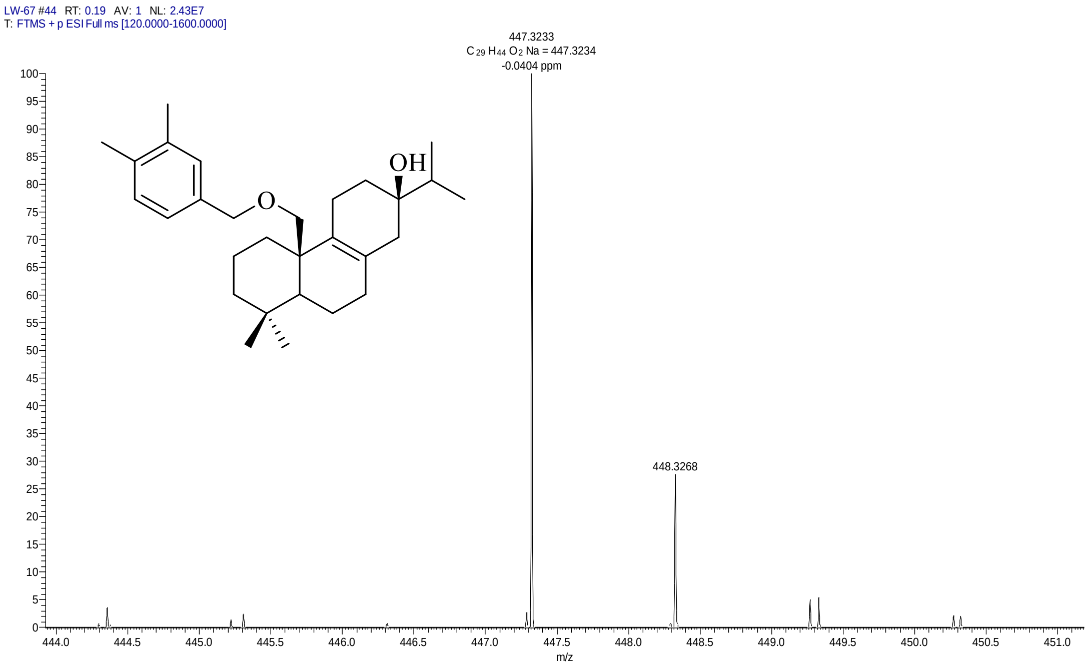

Figure S69. HRMS spectrum of the compound **5h**.

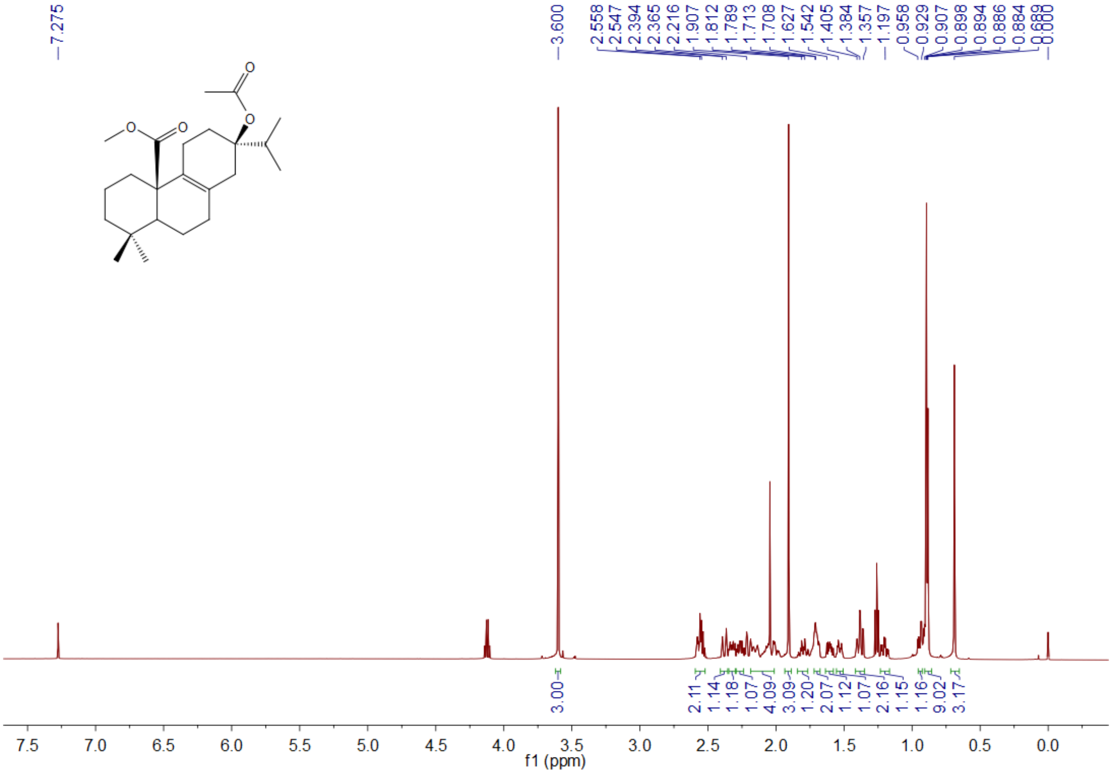

Figure S70. ^{1}H NMR spectrum of the compound **6a**.

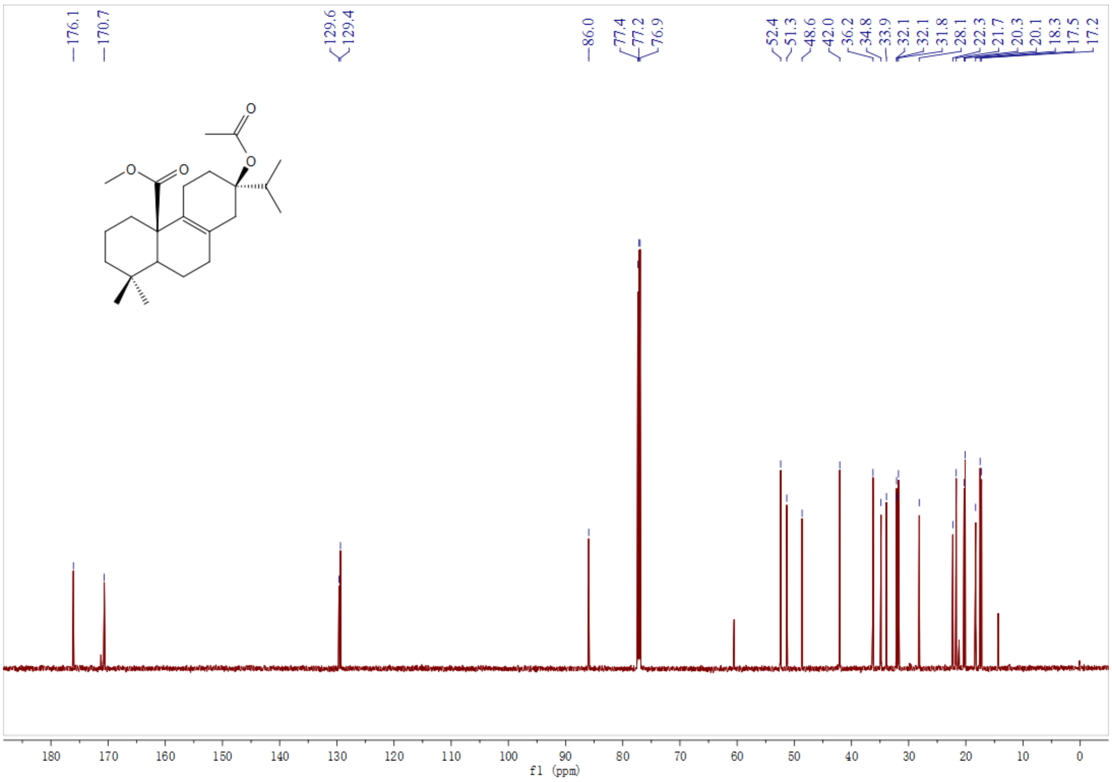

Figure S71. ^{13}C NMR spectrum of the compound **6a**.

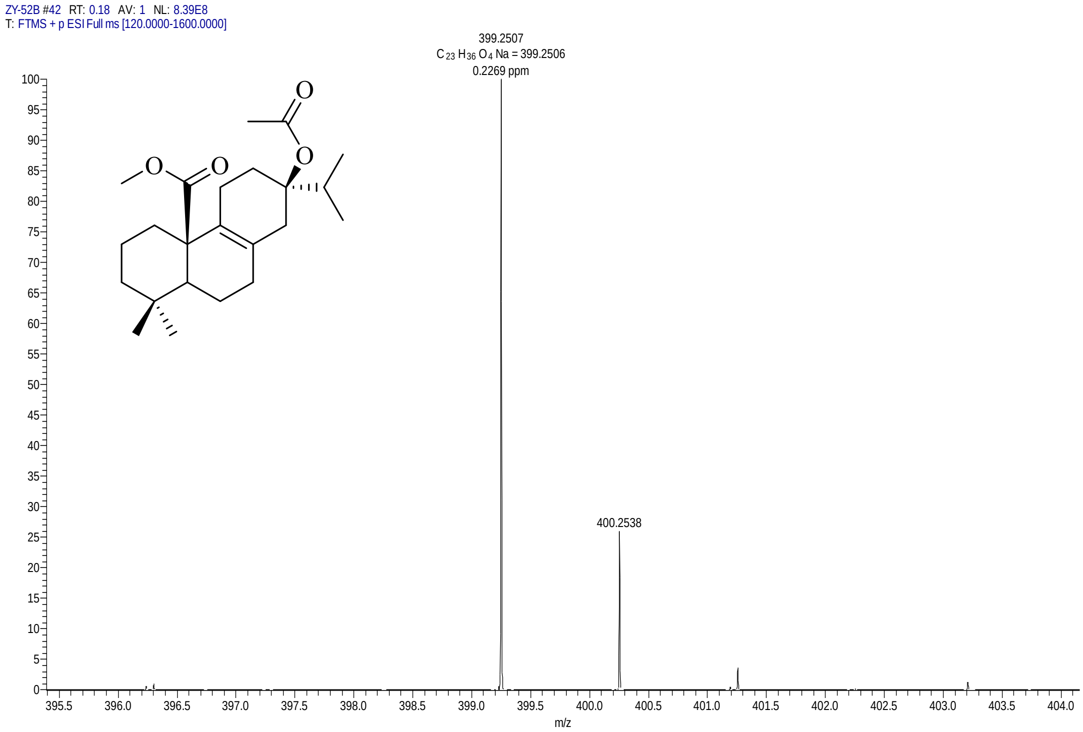

Figure S72. HRMS spectrum of the compound **6a**.

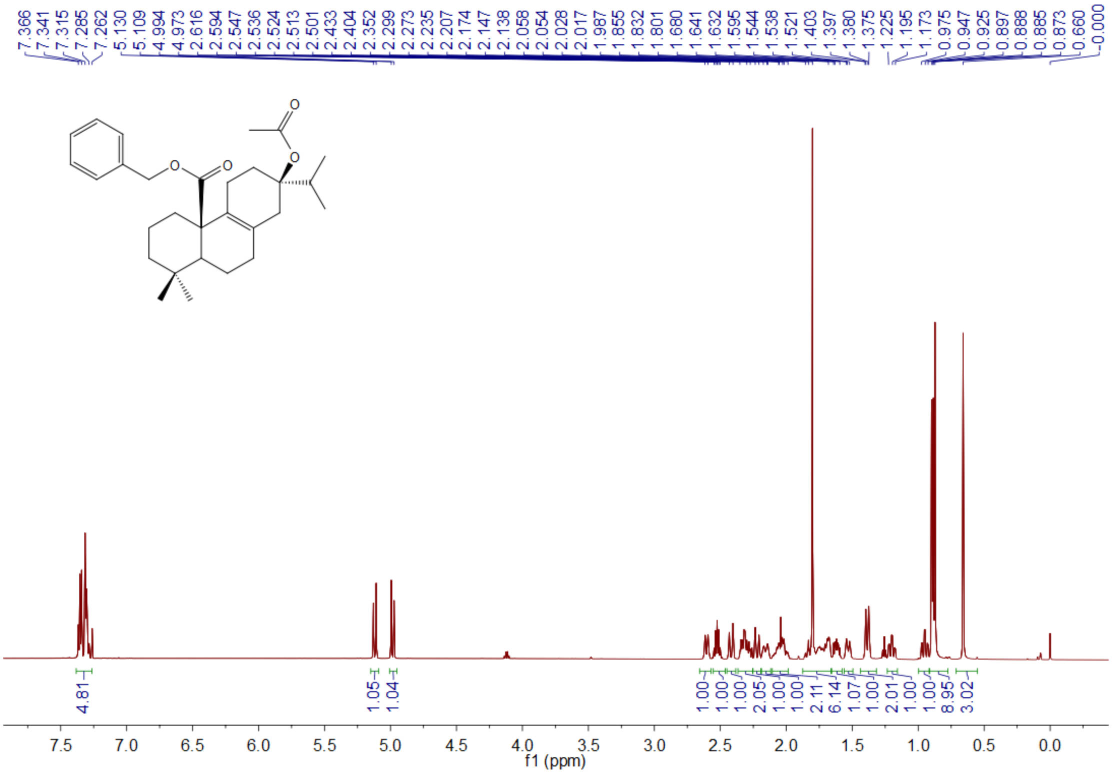

Figure S73. ^{1}H NMR spectrum of the compound **6b**.

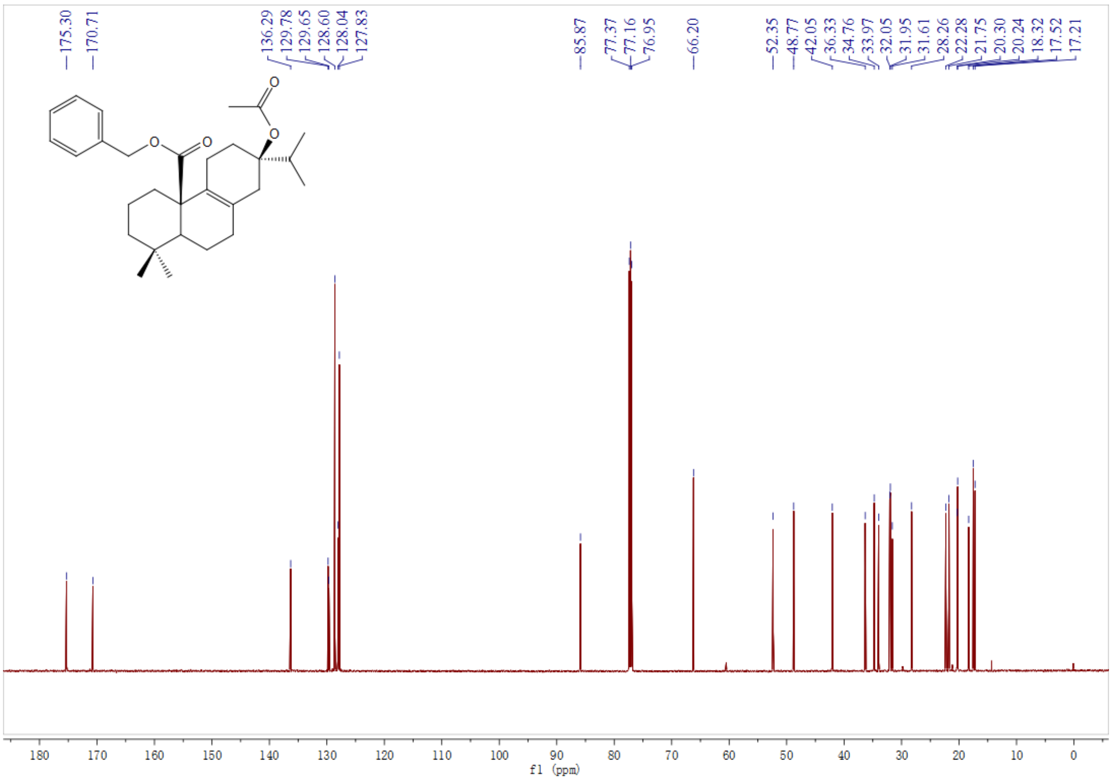

Figure S74. ^{13}C NMR spectrum of the compound **6b**.

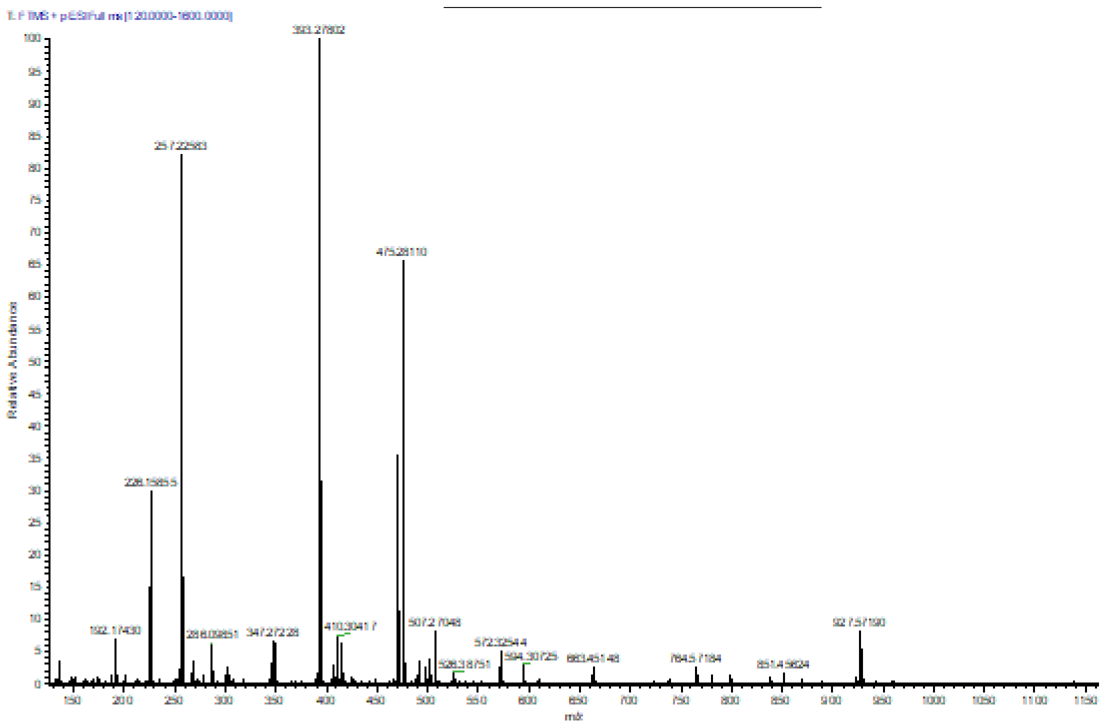

Figure S75. HRMS spectrum of the compound **6b**.

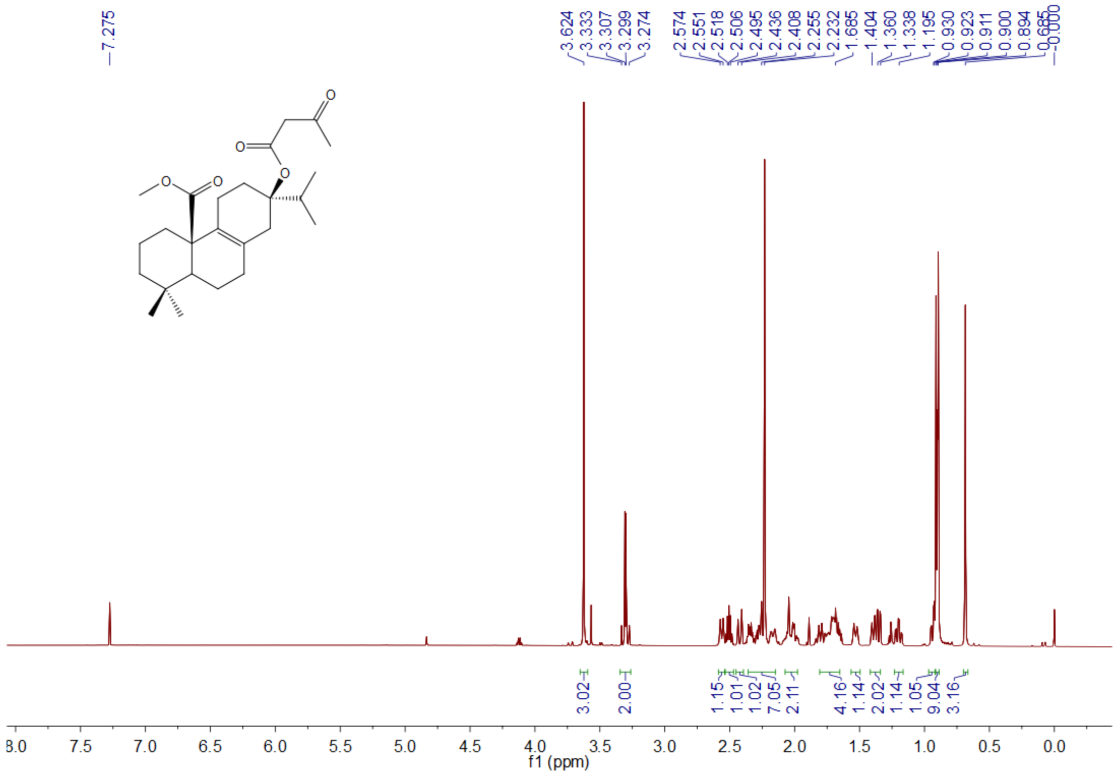

Figure S76. ^{1}H NMR spectrum of the compound **6c**.

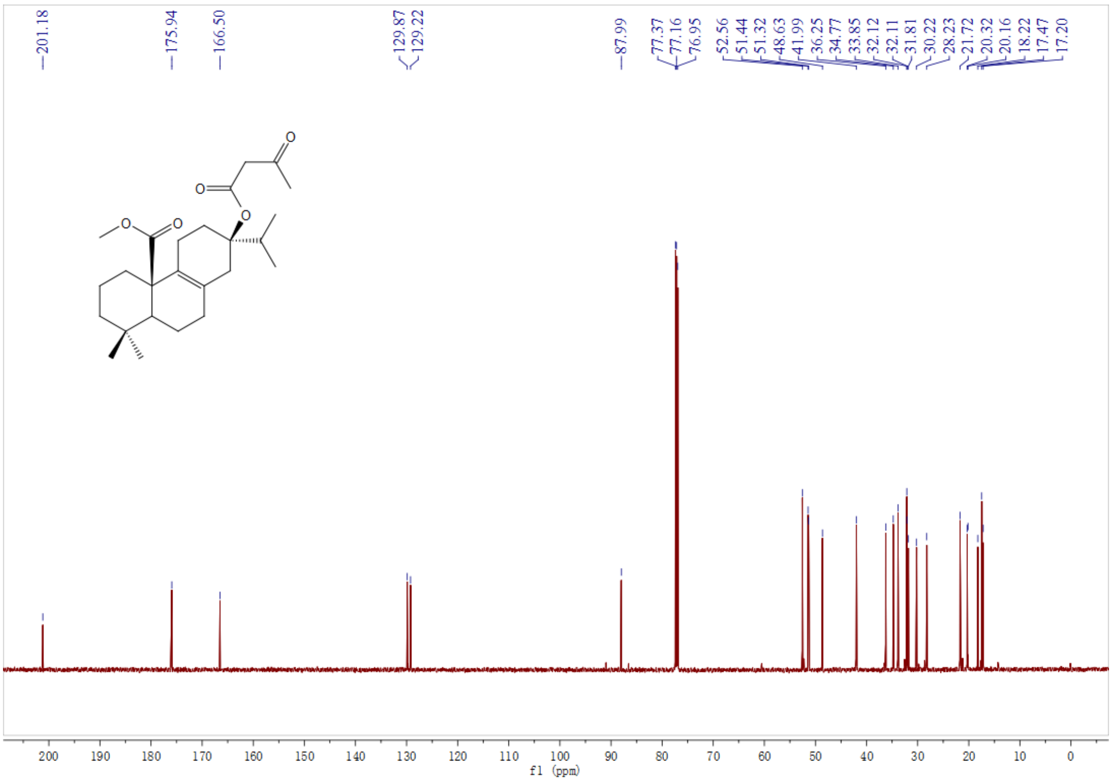

Figure S77. ^{13}C NMR spectrum of the compound **6c**.

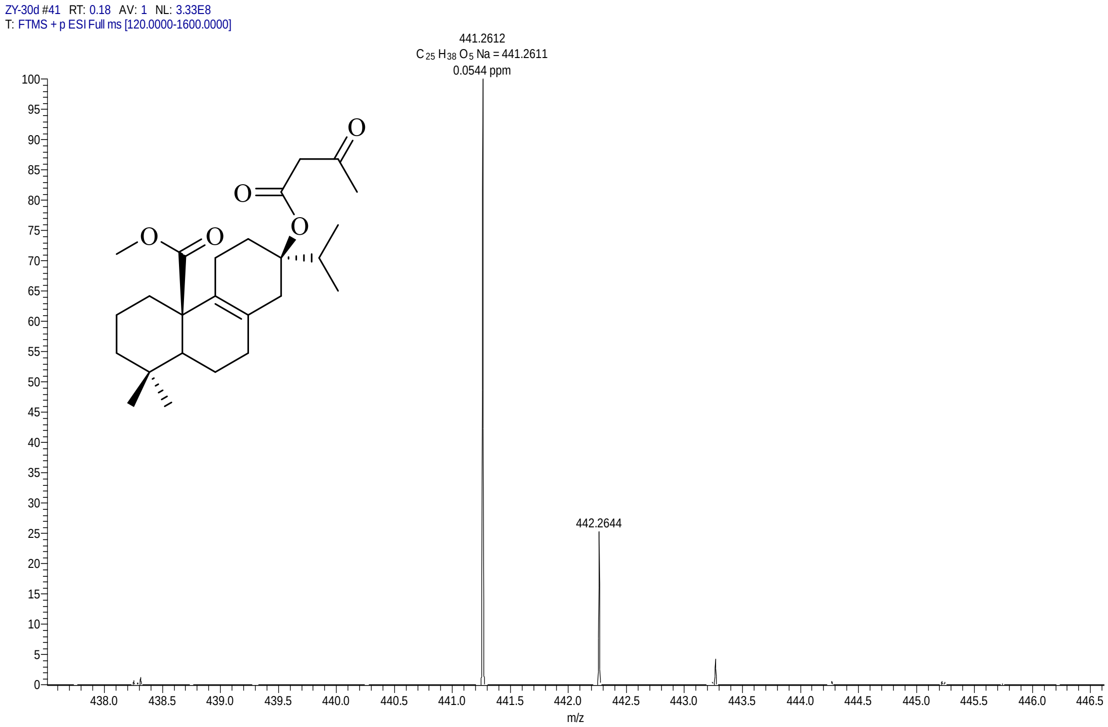

Figure S78. HRMS spectrum of the compound **6c**.
